# A p-n heterojunction sonosensitizer for improved sono-immunotherapy via induction of multimodal cell death mechanisms

**DOI:** 10.7150/thno.106999

**Published:** 2025-01-27

**Authors:** Sijia Wu, Qian Wang, Jun Du, Qingxuan Meng, Yuhao Li, Yuqing Miao, Qing Miao, Jingxiang Wu

**Affiliations:** 1 Department of Anesthesiology, Shanghai Chest Hospital, Shanghai Jiao Tong University, School of Medicine, Shanghai 200030, China.; 2 School of Materials and Chemistry, Institute of Bismuth Science, Shanghai Collaborative Innovation Center of Energy Therapy for Tumors, University of Shanghai for Science and Technology, Shanghai 200093, China.

**Keywords:** Sonodynamic therapy, Heterojunction, Cuproptosis, Ferroptosis, Immunogenic cell death

## Abstract

**Rationale:** Activating a robust immune system is a crucial strategy for combating solid tumors and preventing recurrences. Studies have shown that cuproptosis and the resulting increased reactive oxygen species (ROS) can trigger immunogenic cell death (ICD) and modulate the tumor immune microenvironment, thereby activating systemic immunity. Therefore, for this purpose, it is important to design a multifunctional copper-based nanomaterial.

**Method:** In this study, we developed Bi_2_O_3-X_S_X_-CuS p-n heterojunction nanoparticles (BCuS NPs) designed to stimulate systemic immune responses and effectively suppress both dormant and recurrent tumors. BCuS nanoparticles were characterized using transmission electron microscopy, X-ray diffraction, and other methods. In addition, the sonodynamic and chemodynamic properties of BCuS were intensively studied by various experimental methods. We identified the mechanisms by which BCuS induced multiple paths of cell death, by using *in vitro* experiments, including immunofluorescence assays, western blotting, and cell flow cytometry. In addition, we used mouse orthotopic and distal tumor models and RNA sequencing to evaluate the efficacy of combination therapy.

**Results:** The results showed that BCuS produced a Fenton-like reaction in an acidic environment and induced the production of highly toxic ROS during ultrasound treatment. *In vitro* studies further showed that BCuS induced the occurrence of cuproptosis and ferroptosis, and stimulated ICD in combination with ROS, thereby effectively reversing the immunosuppression of the tumor microenvironment, and improving the sensitivity of immunotherapy. As demonstrated by *in vitro* studies, *in vivo* experiments also confirmed the enhanced effects of combination therapy.

**Conclusion:** The BCuS sonosensitizer showed sonodynamic therapy effects, including inhibition of tumor growth in combination with multiple cell death modalities. These findings provide a novel method for using nanomaterials for multimodal combination cancer therapy.

## Introduction

In recent years, clinical tumor immunotherapy has made significant progress. However, effectively eliciting a sustained systemic immune response remains a critical challenge 1. The variability in immunotherapy outcomes is primarily due to the lack of infiltrating immune cells and the accumulation of immunosuppressive cells within the tumor microenvironment (TME) 2. Consequently, researchers are striving to enhance the intensity of immune responses to improve the efficacy of immunotherapy by targeting various modes of cell death 3.

The use of metal ion metabolism imbalances to mediate cell death for cancer treatment has been extensive. Among these, cuproptosis has emerged as a possible form of cancer cell death. This process is initiated by the direct interaction of copper ions with the lipoic acid component of the tricarboxylic acid (TCA) cycle, which results in the loss of Fe-S cluster proteins, subsequent protein toxicity, and ultimately cell death 4,5. Studies have demonstrated that cuproptosis can trigger immunogenic cell death (ICD), which enhances immune cell infiltration into tumor sites, modulates the tumor immune microenvironment, and activates systemic immunity 6-8. During ICD, dying tumor cells release damage-associated molecular patterns (DAMPs), including the surface exposure of calreticulin (CRT) and the extracellular secretion of heat shock proteins, high-mobility group box 1 (HMGB1) protein, and adenosine triphosphate (ATP) 9,10. These DAMPs serve as danger signals that recruit and activate antigen-presenting cells, facilitate the maturation of dendritic cells (DCs), and promote infiltration of cytotoxic T lymphocytes (CD8^+^ T cells) into tumors. This process effectively counteracts the immunosuppressive nature of the TME and enhances the efficacy of immunotherapy 10,11. However, the relatively low levels of copper ions in tumor tissues present a challenge for inducing cuproptosis. Therefore, the development of nanomaterials capable of delivering additional copper ions to tumors is crucial.

Cells absorb and utilize Cu^+^, but Cu^+^ is unstable and highly toxic to normal cells 12. To safely and effectively introduce copper ions into the body, an alternative strategy involves using stable, low-toxicity Cu^2+^, which can be reduced to Cu^+^ *in vivo* to induce cuproptosis. Copper sulfide nanoparticles (CuS NPs) are characterized by their small particle size, ease of modification, and low toxicity. They have been widely used in biomedical applications 13,14. However, the performance of pure CuS is limited due to rapid electron-hole recombination and low quantum yield 15. Various strategies have been used to address these limitations, such as constructing heterojunctions and introducing surface defects or vacancies 16. Among these, constructing heterojunctions is a cutting-edge strategy that can improve charge separation efficiency, suppress carrier recombination, and spontaneously induce unique physicochemical properties (including interface coupling, built-in electric fields, and space charge regions) to surpass intrinsic activity limitations 17. Compared to p-p heterojunctions, p-n heterojunctions provide significant advantages due to differences in Fermi levels and electrostatic attractions between different carrier species, which enhance charge separation 18. Therefore, combining p-type semiconductor CuS with n-type semiconductors to form p-n heterojunctions is a promising approach.

Recently, bismuth-based nanomaterials, such as n-type semiconductor S-doped Bi_2_O_3_ (Bi_2_O_3-X_S_X_), have attracted significant attention in cancer therapy and other fields 19,20. The porous, stable, and tunable bandgap properties of Bi_2_O_3-X_S_X_ make it an ideal substrate for heterojunctions. Additionally, due to the intrinsic properties of bismuth ions, Bi_2_O_3-X_S_X_ can coordinate with glutathione (GSH) to modulate redox levels, thereby inducing oxidative damage in tumors 21,22. Moreover, Bi_2_O_3-X_S_X_ acts as a low-toxicity sonosensitizer, which can generate reactive oxygen species (ROS) upon ultrasound (US) stimulation, facilitating sonodynamic therapy (SDT) for tumor treatment. As a noninvasive treatment, SDT uses mechanical waves with frequencies well beyond the audible range, thereby overcoming the limitation of photothermal therapy in treating deeper tumors, due to its limited penetration depth 23. Additionally, SDT provides benefits such as noninvasiveness, safety, ease of control, and strong tissue penetration, making it a promising approach for cancer treatment 24,25.

**Scheme 1:**
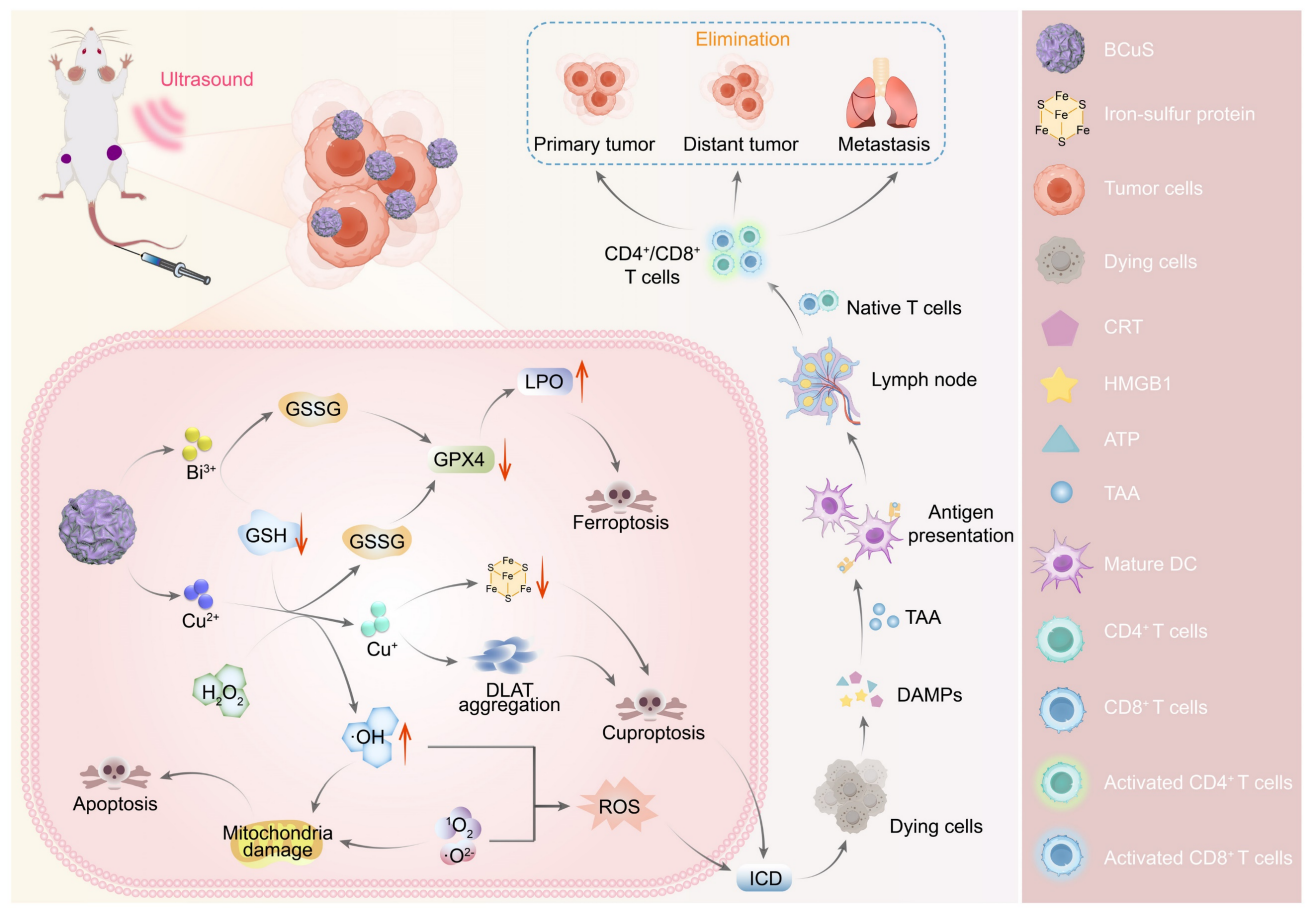
Diagram illustrating the mechanism of BCuS heterojunction construction, US-catalyzed ROS generation, cuproptosis, ferroptosis, and the activation of the immune system through ICD for synergistic tumor therapy.

Based on these properties, this study used Bi_2_O_3-X_S_X_ as an n-type semiconductor and electrostatically adsorbed p-type semiconductor CuS onto its surface to construct the Bi_2_O_3-X_S_X_-CuS (BCuS) p-n heterojunction. This heterojunction was designed to synergistically enhance SDT in combination with cuproptosis and ferroptosis, to coordinate and amplify ICD, thereby changing immunogenicity (Scheme 1). The Fermi level difference between Bi_2_O_3-X_S_X_ and CuS, along with the electrostatic attraction between electrons and holes, facilitated charge carrier migration. As a result, BCuS generated ROS during US stimulation, inducing oxidative stress in tumors. Furthermore, formation of the p-n heterojunction led to band bending, which resulted in a cascade of ROS production, elevating ROS levels and inducing apoptosis. Within the TME, BCuS released Cu^2+^ and Bi^3+^ in the presence of excess GSH. In the presence of endogenous hydrogen peroxide (H_2_O_2_), Cu^2+^ underwent a Fenton-like reaction to convert it into highly toxic Cu^+^, to induce cuproptosis. Concurrently, H_2_O_2_ was converted into hydroxyl radicals (•OH), which, in conjunction with Bi^3+^ depleting GSH, led to the inactivation of glutathione peroxidase 4 (GPX4) and induced ferroptosis. The extensive production of ROS and occurrence of cuproptosis in these processes then triggered ICD. Subsequently, dying cancer cells released DAMPs, which promoted DC maturation and T-cell activation, significantly inhibiting both local and distant tumor growth. Overall, the development of this novel p-n heterojunction copper carrier provided a new strategy for multimodal combined treatment of breast cancer.

## Experimental methods

### Materials

Bi(NO_3_)_3_·5H_2_O (99%), NH_4_F (99.99%), Cu(NO_3_)_2_·3H_2_O (99%), Na_2_S·9H_2_O (99%), ethylene glycol (≥ 99.5%), glycerol (99.0%), and polyvinylpyrrolidone K30 (PVP, molecular weight: 40,000) were obtained from Adamas, China. Rhodamine B (RhB) (97%) was purchased from Macklin (China). All reagents were used as received without further purification, and the water utilized was deionized or doubly distilled.

### Synthesis of BiF_3_ nanoparticles

BiF_3_ nanoparticles were synthesized at room temperature using a co-precipitation method. Initially, Bi(NO_3_)_3_·5H_2_O (0.01 mmol) and NH_4_F (0.3 mmol) were dissolved separately in 10 mL and 25 mL of ethylene glycol, respectively, resulting in solution 1 and solution 2. Solution 2 was then rapidly added to solution 1 while stirring at 1,000 rpm for 60 seconds (s). After stirring was halted, 35 mL of deionized water was introduced to terminate the reaction. The mixture was centrifuged, and the resulting precipitate was washed three times with deionized water. The precipitate was subsequently dispersed in deionized water and stored at 4 °C.

### Synthesis of BCuS nanoparticles

In the first step, 350 mg of PVP and 70 mg of Cu(NO_3_)_2_·3H_2_O were each dissolved in deionized water. At room temperature, the Cu(NO_3_)_2_ solution was added dropwise to the PVP solution while stirring for 10 minutes (min). Following this, the resulting Cu^2+^-PVP solution was combined with a solution containing 70 mg of BiF_3_ and stirred for an additional 30 min. Na_2_S·9H_2_O was dissolved in deionized water and added dropwise to the mixture, stirring for 5 min. The entire solution was then heated to 90 °C and stirred for an additional 50 min. After the heating was stopped, the solution was allowed to cool to room temperature and then centrifuged. The precipitate was alternately washed with water and ethanol three times, collected, and dispersed in deionized water for storage at 4 °C.

### Detection of ROS generation

To detect the generation of superoxide anion radicals (•O_2_^-^) and singlet oxygen (^1^O_2_), BCuS (50 µg mL^-1^, 2 mL) was mixed with 1,3-diphenylisobenzofuran (DPBF, 99%, Adamas, China, 40 µM) and subjected to US stimulation (1 W cm^-2^). The changes in the absorbance of DPBF at 421 nm were monitored at various times. The results were normalized using the formula: A = (A_tmin_ - A_m_)/(A_0min_ - A_m_). Here, A_0min_ and A_tmin_ represent the initial absorbance before US stimulation and the absorbance at time t during the treatment, respectively. A_m_ denotes the absorbance of BCuS at 421 nm.

To further investigate the generation of •O_2_^-^, BCuS (50 µg mL^-1^, 2 mL) was mixed with nitroblue tetrazolium chloride (NBT, ≥ 98%, Adamas, China, 20 µM), and the procedure was repeated. The absorbance of NBT at 260 nm was normalized using the previously mentioned formula, where A_m_ represents the absorbance of BCuS at 260 nm. For the assessment of ^1^O_2_ generation, BCuS (50 µg mL^-1^, 2 mL) was mixed with 9,10-diphenylanthracene (DPA, 97%, Adamas, China, 50 µM), and the procedure was repeated. The absorbance of DPA at 400 nm was normalized using the same formula, with A_m_ indicating the absorbance of BCuS at 400 nm. In addition, dihydrorhodamine 123 (DHR123, KeyGEN, China) was used to detect the production of •O_2_^-^. BCuS (50 μg mL^-1^, 2 mL) was mixed with DHR123 (5 μM) and US (1 W cm^-2^). The changes in fluorescence intensity of DHR123 at 526 nm were detected at different time points.

### Detection of chemodynamic process

Different concentrations of BCuS (25, 50, 100, 150, 200 µg mL^-1^), H_2_O_2_ (0.5 mM), and 3,3´,5,5´-tetramethylbenzidine (TMB, 98%, Adamas, China, 0.8 mM) were mixed in phosphate buffer solutions (PBS, 3 mL) at different pH values (7.4 or 5.5). The mixtures were incubated at room temperature for 30 min, and the absorbance of oxidized TMB (ox-TMB) was measured at 648 nm and 895 nm to evaluate the chemical kinetics of BCuS at various concentrations. Additionally, to investigate the impact of copper ion consumption on GSH (99%, Adamas, China) and its effect on the chemodynamic process, different concentrations of BCuS (25, 50, 100, 150, 200 µg mL^-1^), H_2_O_2_ (0.5 mM), GSH (1 mM), and TMB (0.8 mM) were mixed in PBS (3 mL) at different pH values (7.4 or 5.5). The mixtures were incubated at room temperature for 30 min, and the absorbance of ox-TMB was measured at 648 nm and 895 nm to assess the chemodynamic process of BCuS in the presence of GSH.

BCuS (50 µg mL^-1^), H_2_O_2_ (0.5 mM), and TMB (0.8 mM) were mixed in PBS (pH = 5.5, 3 mL). The absorbance of ox-TMB at 648 nm and 895 nm was recorded at different time intervals. This data was used to determine the chemodynamic process of the same concentration of BCuS over time.

### Consumption of GSH *in vitro*

The samples were divided into eight groups: (1) pH = 5.5 control, (2) pH = 7.4 control, (3) pH = 5.5 + US, (4) pH = 7.4 + US, (5) pH = 5.5/BCuS, (6) pH = 7.4/BCuS, (7) pH = 5.5/BCuS + US, and (8) pH = 7.4/BCuS + US. In all groups, the concentration of GSH was maintained at 10 mM. For groups (5), (6), (7), and (8), the concentration of BCuS was set at 100 µg mL^-1^. Groups (3), (4), (7), and (8) were subjected to US treatment (1 W cm^-2^) for 2 min, after which all groups were incubated at room temperature for 30 min. Subsequently, the supernatant from each group was collected by centrifugation and transferred to a 96-well plate. 5,5'-dithiobis(2-nitrobenzoic acid) (DTNB, Beyotime Biotechnology, China, 10 µL of a 3 mg mL^-1^ solution) was rapidly added to each well, mixed thoroughly, and the absorbance at 414 nm was measured using a microplate reader (BioTek Cytation 3, USA).

### Cell culture

The 4T1 mouse breast cancer cells and 293T human embryonic kidney cells were obtained from the Shanghai Institute of Life Sciences, Chinese Academy of Sciences. The 4T1 cells were cultured in RPMI-1640 medium (Adamas, China) supplemented with 10% fetal bovine serum (Adamas, China) and 1% penicillin-streptomycin (Adamas, China). The cells were maintained in a cell culture incubator (37 °C, 5% CO_2_, Thermo Fisher, USA).

### Cell groups and treatment conditions

The 4T1 cells were seeded into culture dishes at a specific density and incubated for 24 hours (h). Four experimental groups were established: (1) Control (cells with no treatment), (2) US (cells treated with US only), (3) BCuS (cells incubated with BCuS only), and (4) BCuS + US (cells incubated with BCuS and then treated with US). The US treatment was administered at a power of 0.7 W cm^-2^ for 5 min. The concentration of BCuS was 55 µg mL^-1^.

### Cell viability assessment

Cell viability was evaluated using the Cell Counting Kit-8 (CCK-8, Beyotime, China). In addition to the four basic experimental groups, four additional groups were included: (5) H_2_O_2_ at pH 7.4 (cells treated with H_2_O_2_ only, with H_2_O_2_ concentrations of 0, 10, 25, 50, 100, and 150 µM), (6) H_2_O_2_ at pH 5.5 (cells treated with H_2_O_2_ only, with H_2_O_2_ concentrations of 0, 10, 25, 50, 100, and 150 µM), (7) H_2_O_2_ + BCuS at pH 7.4 (cells treated with H_2_O_2_ and BCuS, with H_2_O_2_ concentrations of 0, 10, 25, 50, 100, and 150 µM, and BCuS at 55 µg mL^-1^), and (8) H_2_O_2_ + BCuS at pH 5.5 (cells treated with H_2_O_2_ and BCuS, with H_2_O_2_ concentrations of 0, 10, 25, 50, 100, and 150 µM, and BCuS at 55 µg mL^-1^). 4T1 and 293T cells were seeded into two 96-well plates at a density of 5,000 cells per well and incubated for 24 h. BCuS (55 µg mL^-1^) was then dispersed in RPMI-1640 and added to groups (3) and (4), which were incubated for an additional 24 or 48 h. Cells in groups (2) and (4) were subjected to US stimulation and then incubated for 6 h. Finally, after washing away dead cells with PBS, CCK-8 solution was added and incubated for 40 min. Absorbance at 450 nm was measured using a microplate reader. Cell viability was calculated using the following formula: Cell viability (%) = Absorption value of different treatment groups/Mean absorption value of control × 100%.

### Flow cytometry for apoptosis detection

Apoptotic cells were detected using an apoptosis detection kit (Annexin V-FITC/PI, Beyotime, China). 4T1 cells were seeded into 6-well plates at a density of 1×10^5^ cells per well and incubated for 24 h. BCuS (55 µg mL^-1^) was then dispersed in RPMI-1640 and added to groups (3) and (4), which were further incubated for 24 h. Cells in groups (2) and (4) were subjected to US stimulation and then incubated for an additional 12 h. Cells were then trypsinized, centrifuged, and washed twice with PBS. Annexin V-FITC and PI were added according to the manufacturer's instructions, and the cells were incubated in the dark for 30 min. Apoptosis was assessed using flow cytometry (BD Melody, USA).

### Detection of CRT exposure and HMGB1 release

4T1 cells were seeded into 6-well plates at a density of 1×10^5^ cells per well and incubated for 24 h. BCuS (55 µg mL^-1^) was then dispersed in RPMI-1640 and added to groups (3) and (4), which were further incubated for 24 h. Cells in groups (2) and (4) were subjected to US stimulation and incubated for an additional 6 h. Cells were then fixed with 4% paraformaldehyde for 30 min. After washing with PBS, cells were permeabilized with 0.3% Triton X-100 for 20 min, followed by blocking with 3% BSA for 2 h. Cells were then incubated overnight at 4 °C with anti-CRT antibody and anti-HMGB1 antibody. Finally, after incubation with a secondary antibody (anti-rabbit 488) for 2 h, cells were stained with DAPI for 15 min. CRT exposure and HMGB1 release were observed using an inverted fluorescence microscope.

### Detection of ATP release

ATP release was quantified using an ATP detection kit (Beyotime, China). 4T1 cells were plated in 6-well plates at a density of 1×10^5^ cells per well and incubated for 24 h. BCuS (55 µg mL^-1^) was then dispersed in RPMI-1640 and added to groups (3) and (4) for an additional 24-hour incubation. Groups (2) and (4) underwent US stimulation and were incubated for another 12 h. Subsequently, the ATP detection reagent was mixed with the cell culture supernatant as per the manufacturer's instructions and added to a 96-well plate. ATP release was quantified using a microplate reader.

### Establishment of tumor models

A 4T1 tumor model was established in 6-week-old female Balb/c mice, obtained from the Experimental Animal Management Department of the Shanghai Institute of Planned Parenthood Research, with approval from the Shanghai Quality Supervision and Inspection Institute of Laboratory Animals (SCXK 2023-0009). All animal experiments were conducted at Shanghai Ruetai Mos Biological Technology Co., Ltd., authorized by the Shanghai Science and Technology Commission (SYXK 2021-0007), and adhered to the guidelines of the Institutional Animal Care and Use Committee of Shidong Hospital, Shanghai (2023-036-01). For tumor implantation, 4T1 cells (2×10^6^ in 100 µL) were injected subcutaneously into the right hind limb of the mice. Seven days later, a tumor model was similarly established in the left hind limb of the same mice.

### *In vivo* combination therapy

BALB/c mice were randomly divided into four groups: (1) Control (n = 6), (2) US (n = 5), (3) BCuS (n = 5), and (4) BCuS + US (n = 6). BCuS was administered at a concentration of 1 mg mL^-1^ (100 µL), with US power set to 0.7 W cm^-2^ for 5 min. Mice in groups (1) and (2) received tail vein injections of PBS, while those in groups (3) and (4) received BCuS. Twelve hours after the injection, mice in groups (2) and (4) underwent US treatment. Twenty-four hours later, one mouse from each of groups (1) and (4) was randomly euthanized, and tumors were quickly excised for RNA sequencing. Throughout the 14-day treatment period, each mouse received only one treatment. Additionally, tumor volume and mouse body weight were recorded every two days, with tumor volume calculated using the following formula: Tumor volume = Length × Width^2^/2.

After 14 days of treatment, all mice were euthanized, and their tumors were excised, photographed, and weighed. Tumors from each group were prepared into tissue sections and subjected to histological analysis using H&E staining and Ki-67 staining. The lungs from each group were also excised, photographed, and stained with H&E.

### Statistical analysis

Statistical differences were calculated using the T-test with SPSS (Statistical Package for the Social Sciences, Chicago, IL, USA). Significance levels are indicated as * P < 0.05, ** P < 0.01, and *** P < 0.001. Error bars represent the mean ± standard deviation for parallel experiments.

## Results and Discussion

### Preparation and structural characterization

**Figure 1:**
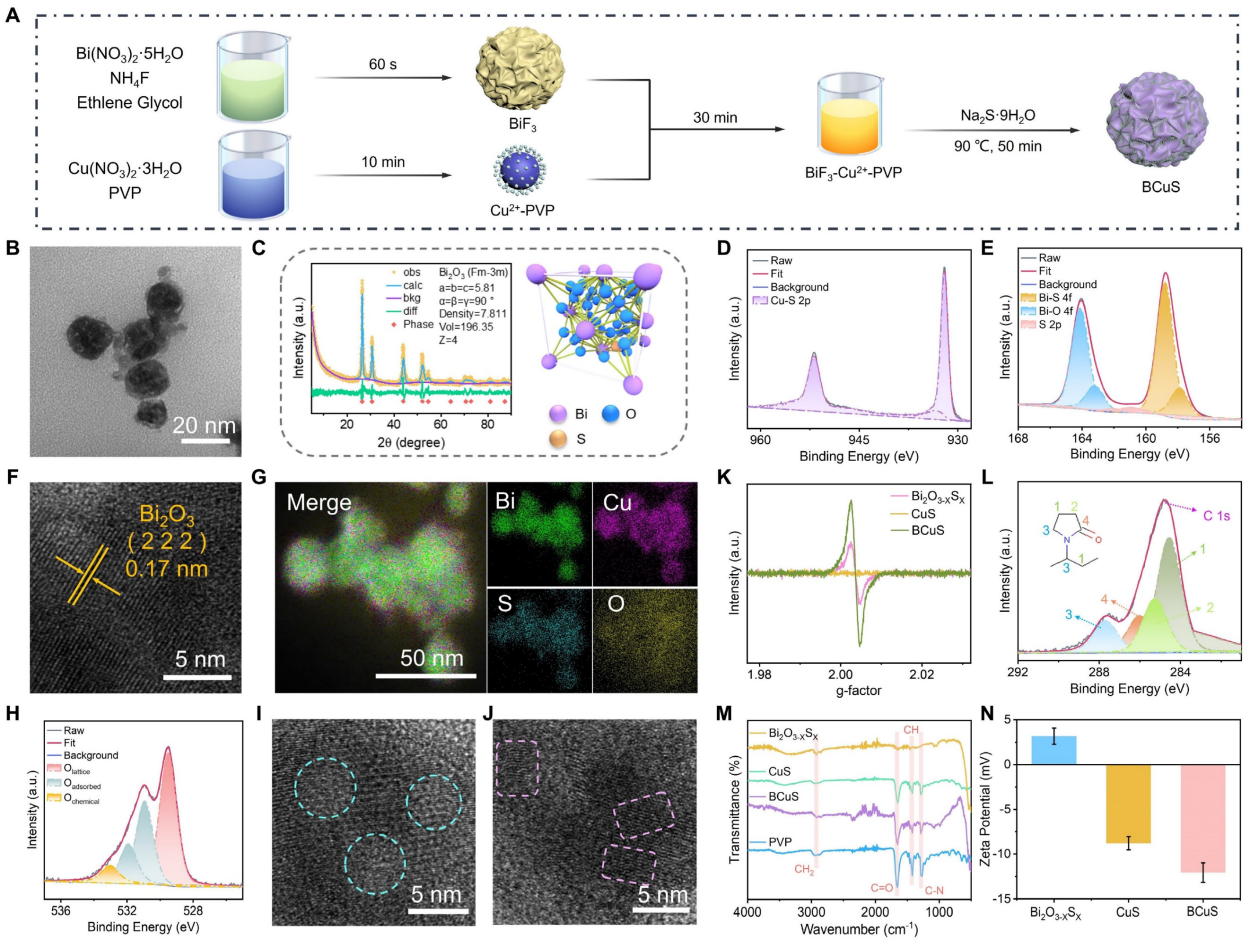
Preparation and characterization of BCuS. (A) Schematic illustration for the synthetic process of BCuS. (B) TEM image of BCuS. (C) XRD pattern of BCuS and refined crystal structure of Bi_2_O_3-X_S_X_. High-resolution XPS spectra of (D) Cu 2p and (E) Bi 4f in BCuS. (F) High-resolution TEM and (G) elemental mapping images of BCuS. (H) High-resolution XPS spectrum of O 1s in BCuS. (I) and (J) are both high-resolution TEM images (blue circles represent amorphous and crystalline states and pink squares represent the distorted lattice). (K) EPR spectra indicate oxygen vacancy. (L) High-resolution XPS spectrum of C 1s in BCuS. (M) FT-IR spectra of Bi_2_O_3-X_S_X_, CuS, BCuS, and PVP. (N) Zeta potential plots.

BCuS nanoparticles were synthesized using a two-step process, as shown in Figure 1A. First, mesoporous BiF_3_ nanoparticles were synthesized with a co-precipitation method 26. Subsequently, a Cu^2+^-PVP complex was formed by combining Cu^2+^ with PVP and then mixing with BiF_3_ nanoparticles. This step facilitated the adsorption of Cu^2+^ onto the surface or into the pores of BiF_3_ through electrostatic interactions. Finally, the BCuS p-n heterojunction was obtained using Na_2_S as the reductant. During this process, Na_2_S simultaneously reduced Cu^2+^ to CuS and facilitated an anion exchange in BiF_3_, leading to the formation of Bi_2_O_3-X_S_X_. Previous studies have reported that bismuth-based dual-anion materials improve photocatalytic carrier separation efficiency 20. Thus, the dual-anion structure may also enhance sonodynamic performance through ion doping that induces asymmetry in the crystal lattice.

Transmission electron microscopy (TEM) images revealed that BCuS exhibited a spherical morphology with an average diameter of 30 ± 10 nm, resembling that of BiF_3_ and Bi_2_O_3-X_S_X_, but with a uniform distribution of CuS nanosheets on the surface (Figures 1B and S1, Supporting Information). X-ray diffraction (XRD) patterns matched well with the cubic phase of Bi_2_O_3_ (Fm-3m, ICSD #38436) (Figure 1C). Rietveld refinement of the XRD data confirmed the successful synthesis of Bi_2_O_3-X_S_X_. The characteristic peaks of CuS were not visible in the XRD pattern due to its small size, which prevented the formation of a crystalline phase (Figure S2). X-ray photoelectron spectroscopy (XPS) further verified the structure of BCuS, showing the presence of Bi, O, S, and Cu (Figure S3). The high-resolution XPS spectra of Cu 2p showed characteristic peaks at 951.98 eV and 932.10 eV, corresponding to Cu-S bonds, indicating the presence of CuS (Figure 1D). In the high-resolution Bi 4f XPS spectrum, peaks at 158.81 eV and 157.84 eV corresponded to Bi-S bonds, further confirming the incorporation of S into Bi_2_O_3_, to form Bi_2_O_3-X_S_X_ (Figure 1E). Semi-quantitative analysis revealed that the ratio of Bi_2_O_3-X_S_X_ to CuS in BCuS was approximately 1.12:1. High-resolution TEM images (Figure 1F) showed a lattice spacing of 0.17 nm, corresponding to the (222) plane of Bi_2_O_3_. Energy-dispersive X-ray spectroscopy mapping showed the uniform distribution of Bi, O, S, and Cu elements (Figure 1G), collectively confirming the successful preparation of BCuS.

Notably, the high-resolution O 1s XPS spectrum revealed a peak at 531.0 eV that was attributed to oxygen vacancies (Figure 1H). TEM images also revealed the interface between crystalline and amorphous structures, as well as distorted lattice fringes (Figures 1I and 1J). Furthermore, BCuS displayed an oxygen vacancy signal (g = 2.003) in electron paramagnetic resonance (EPR) spectra (Figure 1K). Together, these findings suggested the presence of oxygen vacancies in the BCuS p-n heterojunction constructed from Bi_2_O_3-X_S_X_ and CuS, which may have contributed to enhanced semiconductor properties.

Due to the addition of PVP during the *in situ* growth of CuS on the Bi_2_O_3-X_S_X_ surface and within its pores, PVP was present on the surface of BCuS. The high-resolution C 1s XPS spectrum showed characteristic peaks of PVP (Figure 1L), and the absorption band at 1666 cm^-1^ in Fourier-transform infrared (FT-IR) spectra further confirmed the presence of PVP (Figure 1M). Moreover, a change in the zeta potential of BCuS (Figure 1N) provided additional evidence for PVP surface modification, which could improve the compatibility of nanoparticles in blood, and extend their circulation times. Dynamic light scattering was used to evaluate the dispersion of BCuS in different solvents (Figure S4), indicating excellent dispersibility in water, PBS (pH 7.4), and RPMI-1640 medium. In addition, there was no significant increase in the hydrated particle size of BCuS after 1 week of storage in water, PBS, or RPMI-1640 medium, indicating that BCuS had high stability (Figure S5).

### Sonocatalytic properties and mechanism of BCuS

**Figure 2:**
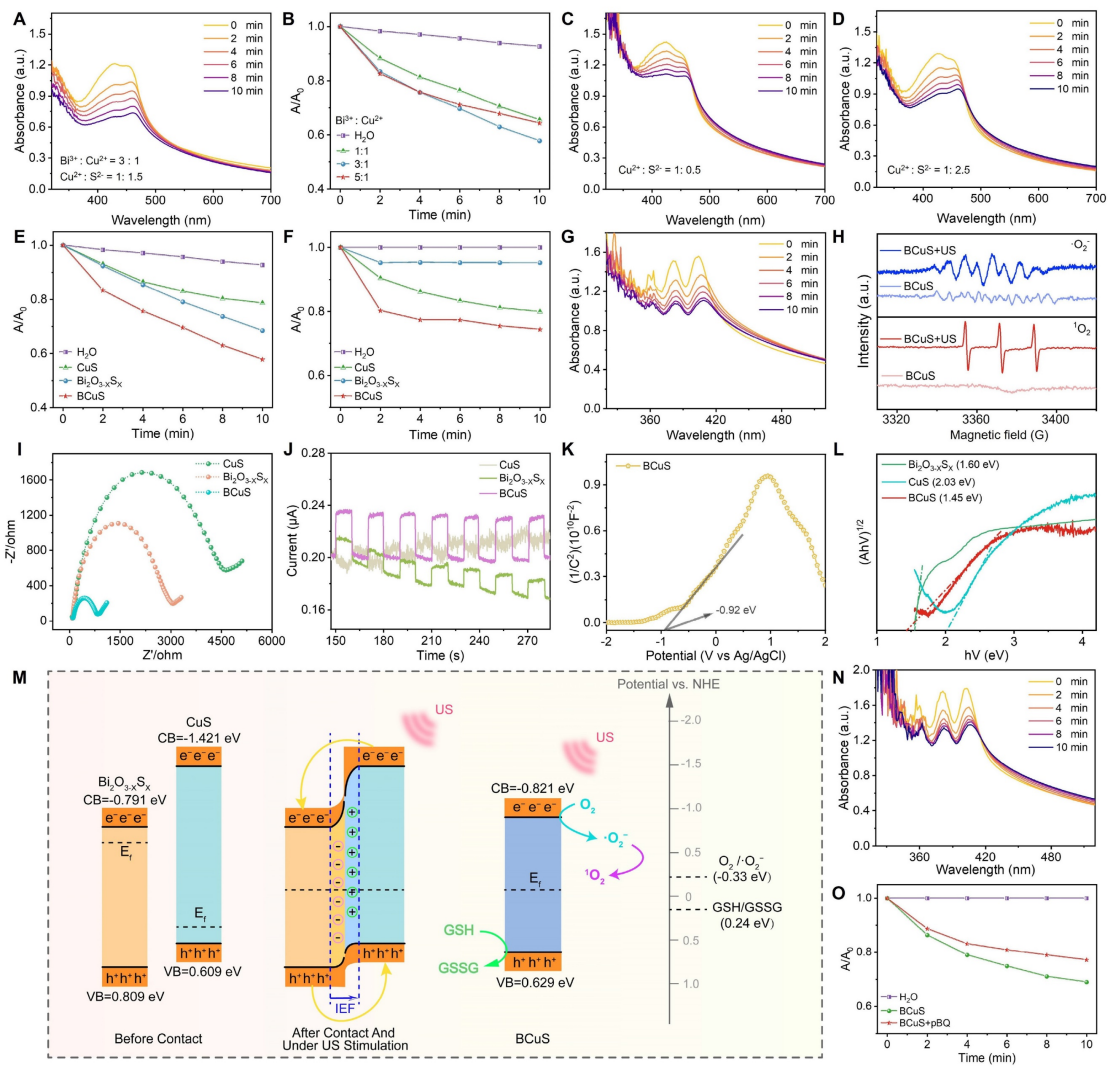
Sonocatalytic performance and mechanism of BCuS. (A) Absorption spectra of BCuS with DPBF under US stimulation over time. (B) Normalized absorption peaks at 421 nm for BCuS in different synthetic feeding ratios mixed with DPBF under US stimulation over time. Absorption spectra of BCuS synthesized in the ratio of Cu(NO_3_)_2_ to Na_2_S were (C) 1:0.5 and (D) 1:2.5 with DPBF under US stimulation over time. Normalized comparison of (E) DPBF characteristic absorption peaks (421 nm) and (F) NBT characteristic absorption peaks (260 nm) for BCuS obtained under final synthesis conditions with those of other groups over time. (G) Absorption spectra of BCuS obtained under final synthesis conditions and DPA under US stimulation over time. (H) EPR spectra confirming the generation of •O_2_^-^ and ^1^O_2_. (I) Electrochemical impedance spectra of Bi_2_O_3-X_S_X_, CuS, and BCuS. (J) Photocurrent response curves of Bi_2_O_3-X_S_X_, CuS, and BCuS. (K) Mott-Schottky plots of BCuS. (L) Solid-state absorption spectra of Bi_2_O_3-X_S_X_, CuS, and BCuS. (M) Sonocatalytic mechanism of BCuS. (N) Absorption spectra of BCuS and DPA mixture under US stimulation over time after adding p-BQ. (O) Normalized comparison of characteristic absorption peaks at 400 nm for BCuS with and without p-BQ in DPA solution.

We investigated the SDT performance of BCuS and identified its underlying mechanism. During synthesis, the SDT performance of BCuS was optimized by adjusting the ratio of starting materials. With fixed reaction time and a constant amount of reductant, we optimized the ratio of BiF_3_ to Cu(NO_3_)_2_ and evaluated the ROS generation using DPBF as the ROS probe. The results showed that the highest ROS yield was achieved at a BiF_3_ to Cu(NO_3_)_2_ ratio of 3:1 (Figures 2A, 2B, and S6). Next, we examined the effect of the Na_2_S amount on ROS yield under this optimized ratio using the DPBF probe. The highest ROS production was obtained when the ratio of Cu(NO_3_)_2_ to Na_2_S was 1:1.5 (Figures 2C, 2D, and S7). Based on these optimized conditions, BCuS was synthesized using a BiF_3_, Cu(NO_3_)_2_, and Na_2_S ratio of 3:1:1.5. The results showed that BCuS exhibited superior sonocatalytic ROS generation capability, when compared to individual Bi_2_O_3-X_S_X_ and CuS at the same concentrations (Figures 2E and S8), indicating that BCuS was effectively activated by US to produce ROS.

To identify the types of ROS generated, NBT and DPA were used as probes for •O_2_^-^ and ^1^O_2_, respectively. When BCuS was mixed with NBT and DPA under US irradiation, the characteristic absorption peaks of both probes decreased significantly, indicating that BCuS continuously catalyzed the generation of •O_2_^-^ and ^1^O_2_ with prolonged US irradiation (Figures 2F, 2G, S9, and S10). In addition, to further validate the •O_2_^-^ produced by BCuS under US stimulation, we used a specialized •O_2_^-^ fluorescent indicator, DHR123. As shown in Figure S11, the fluorescence spectrum of the BCuS group showed a significant upward trend compared with that of the pure water group under US stimulation, indicating the production of •O_2_^-^. This conclusion was further supported by EPR spectra, which showed characteristic peaks corresponding to both types of ROS (Figure 2H).

Because of their sono-electrochemical effect, it has been reported that sonosensitizers absorb energy under US irradiation and induce electron movement 23. To evaluate the charge transfer capability and carrier separation efficiency of the BCuS p-n heterojunction, and to understand its underlying mechanism, we conducted electrochemical tests on Bi_2_O_3-X_S_X_, CuS, and BCuS. Electrochemical impedance spectroscopy results showed that the Nyquist semicircle of BCuS was significantly smaller than those of Bi_2_O_3-X_S_X_ and CuS (Figure 2I), indicating that the formation of the p-n heterojunction reduced the material's impedance and optimized the band structure, thereby enhancing electron-hole transfer efficiency. Moreover, transient photocurrent response measurements showed that BCuS exhibited a significantly higher photocurrent density under US stimulation, when compared with individual Bi_2_O_3-X_S_X_ and CuS (Figure 2J), suggesting that BCuS generated more charge carriers upon US stimulation. In addition, the previously observed oxygen vacancies in BCuS may have resulted in a narrower band gap, which could further enhance charge separation efficiency.

The interfacial behavior of BCuS charge carriers was further assessed using Mott-Schottky analysis. The flat band potentials (E_fb_) of Bi_2_O_3-X_S_X_, CuS, and BCuS were determined to be -0.89 eV, 0.51 eV, and -0.92 eV, respectively, relative to the Ag/AgCl electrode (Figures 2K and S12). Converting these flat band potentials to values relative to the normal hydrogen electrode (NHE) revealed the relationships between E_fb_ and the conduction band (CB) or valence band (VB) 27,28. The CB levels of n-type Bi_2_O_3-X_S_X_ and BCuS were -0.791 eV and -0.821 eV, respectively, while the VB level of p-type CuS was 0.609 eV. Solid-state diffuse reflectance spectroscopy further confirmed the band gaps of these three materials (Figure 2L). These results showed that the band gap of BCuS (1.45 eV) was significantly lower than those of Bi_2_O_3-X_S_X_ (1.60 eV) and CuS (2.03 eV), suggesting that formation of a p-n heterojunction reduced the band gap, enabling BCuS to effectively respond to US stimulation and generate more charge carriers.

Based on these results, we proposed the mechanism of the sonocatalytic activity of the BCuS p-n heterojunction (Figure 2M). When p-type CuS and n-type Bi_2_O_3-X_S_X_ formed a p-n heterojunction, electrons in BCuS spontaneously migrated from Bi_2_O_3-X_S_X_ to CuS, due to the lower Fermi level and larger band gap of CuS. This migration continued until the Fermi level reached equilibrium. Consequently, most electrons in the n-type semiconductor migrated to the surface of the p-type semiconductor. This continuous charge transfer process established an internal electric field at the Bi_2_O_3-X_S_X_/CuS interface, directed from Bi_2_O_3-X_S_X_ to CuS, providing a directional pathway for sustained electron flow. Under US stimulation, photoacoustic electrons spontaneously accumulated in Bi_2_O_3-X_S_X_, while photo-generated holes migrated to CuS. During this process, the band gap and electron affinity of the semiconductors remained unchanged, resulting in downward band bending for the p-type semiconductor and upward band bending for the n-type semiconductor, facilitating electron-hole tunneling. Additionally, the potential of BCuS reached the threshold for •O_2_^-^ generation (O_2_/•O_2_^-^ = -0.33 eV relative to NHE), which verified the previous ROS detection results. Some studies suggest that sonocatalysis favors the occurrence of cascade reactions, leading us to hypothesize that the generated ^1^O_2_ may have originated from •O_2_^-^. The addition of the p-benzoquinone •O_2_^-^ scavenger to the DPA solution used for detecting ^1^O_2_ resulted in a decreased DPA absorption peak during US irradiation (Figures 2N and 2O), confirming the occurrence of a ROS cascade reaction in BCuS, which promoted the production of highly toxic ^1^O_2_. In addition, part of the ^1^O_2_ was derived from the direct conversion of O_2_ during US irradiation. Therefore, formation of a p-n heterojunction was beneficial for charge carrier separation, which increased the sonocatalytic performance.

### Acid-responsive chemodynamic therapy and multiple TME modulation

**Figure 3:**
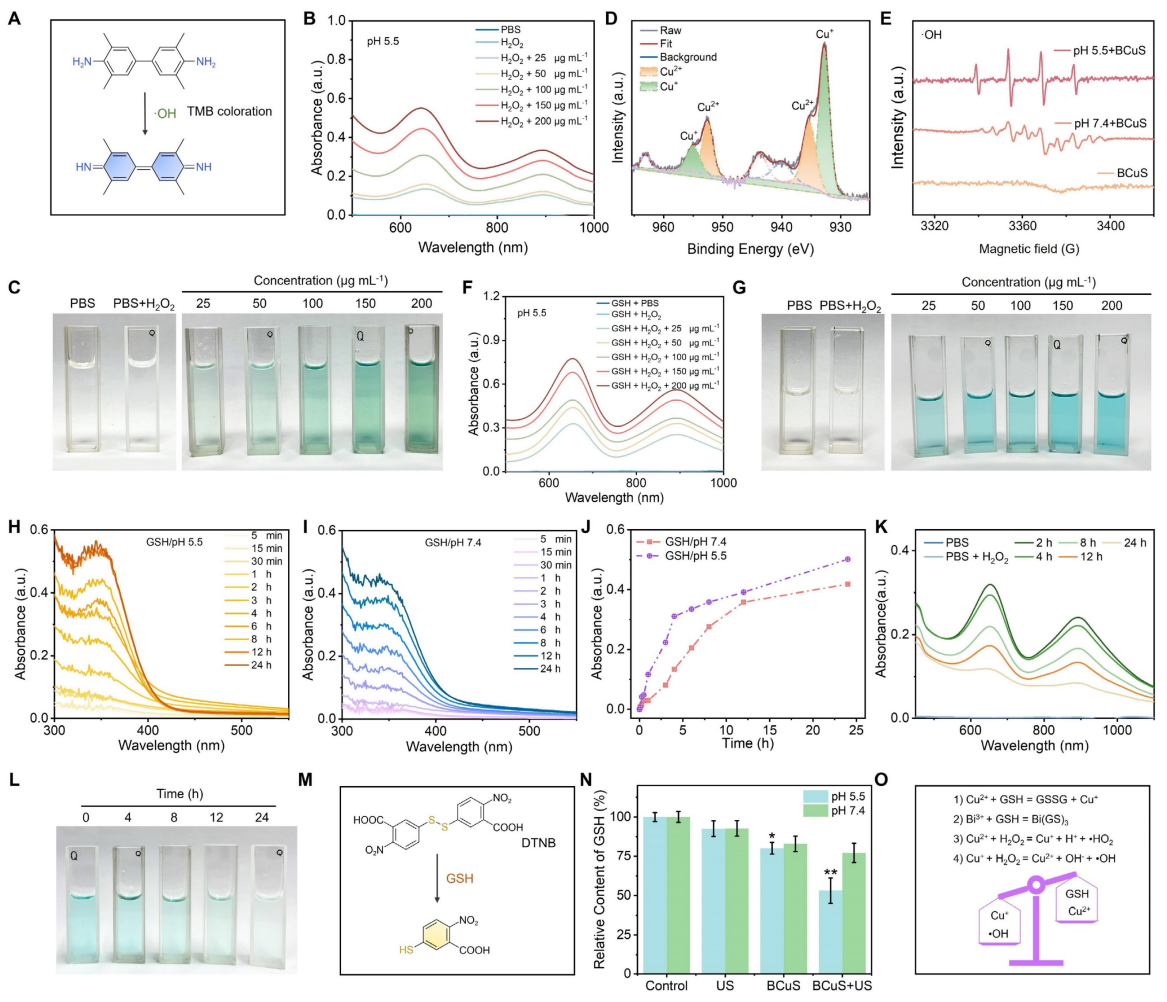
CDT performance of BCuS and strategies for multi-faceted regulation of the TME. (A) Reaction of TMB with •OH to form the blue products (ox-TMB) (•OH detection). (B) Absorption spectra of ox-TMB in the mixed solution as a function of BCuS concentration under pH 5.5/H_2_O_2_ conditions. (C) Digital photographs showing the color change of ox-TMB under pH 5.5/H_2_O_2_ conditions. (D) High-resolution XPS spectrum of Cu 2p. (E) EPR spectra of ŸOH generated by BCuS under different conditions. (F) Absorption spectra of ox-TMB in the mixed solution as a function of BCuS concentration under pH 5.5/GSH/H_2_O_2_ conditions. (G) Digital photographs showing the color change of ox-TMB under pH 5.5/GSH/H_2_O_2_ conditions. Time-dependent changes in the absorption peak at 348 nm of the supernatant after incubating BCuS under (H) pH 5.5/GSH and (I) pH 7.4/GSH conditions. (J) Normalized comparison of absorption peaks at 348 nm for the supernatants from (H) and (I). (K) Time-dependent changes in the absorption spectra of ox-TMB in the mixed solution after incubating BCuS under pH 5.5/GSH/H_2_O_2_ conditions and (L) digital photographs showing the color change of ox-TMB. (M) DTNB with GSH to form the yellow products (GSH detection). (N) Relative GSH content under different conditions. (O) Schematic diagram of the mechanism by which BCuS develops CDT and regulates TME under acidic conditions. Data are presented as mean ± standard deviation (n = 4) (* P < 0.05, ** P < 0.01, *** P < 0.001).

The presence of copper ions in BCuS suggested a Fenton-like reaction. We therefore used TMB as a probe to detect •OH. As shown in Figures 3A and 3B, neither TMB alone nor TMB mixed with H_2_O_2_ exhibited the characteristic absorption peak of ox-TMB. However, when BCuS was mixed with TMB in the presence of acidic (pH 5.5) H_2_O_2_, a characteristic absorption peak of ox-TMB appeared at 650 nm. The intensity of this peak increased with higher BCuS concentrations, indicating that BCuS catalyzed the production of •OH from H_2_O_2_. Furthermore, the color of the TMB solution changed from colorless to blue as the BCuS concentration increased, further showing the generation of ox-TMB (Figure 3C). However, under neutral conditions (pH 7.4), even in the presence of H_2_O_2_, the mixture of BCuS and TMB did not exhibit a characteristic absorption peak at 650 nm (Figure S13), suggesting that BCuS had a slower rate of decomposition during neutral conditions, which was unfavorable for initiating a Fenton-like reaction. We therefore used XPS to determine the oxidation state of copper ions in BCuS in acidic H_2_O_2_. High-resolution Cu 2p XPS spectrum revealed the coexistence of Cu^2+^ and Cu^+^, indicating the occurrence of a Fenton-like reaction (Figure 3D). EPR spectra further confirmed the generation of •OH (Figure 3E). These results showed that BCuS only catalyzed the production of ROS in mildly acidic environments, to induce chemodynamic therapy (CDT) against tumors, while no ROS was generated under non-acidic conditions.

Previous studies have also reported that Cu^2+^ reacts with GSH to produce Cu^+^ and oxidized glutathione, thereby enhancing CDT performance 29. To determine whether the presence of GSH increased •OH production, we mixed different concentrations of BCuS with TMB and added acidic GSH and H_2_O_2_. The results showed that under these conditions, the characteristic absorption peak of ox-TMB significantly increased, indicating that BCuS exhibited enhanced CDT performance in the GSH-enriched acidic TME (Figure 3F). The results shown in Figure 3G further supported this conclusion.

In addition to Cu^2+^, Bi^3+^ has also been reported to interact with GSH, leading to GSH depletion and inducing the degradation of nanomaterials. We examined the degradation kinetics of BCuS at different pH values. Figures 3H and 3I show that in the presence of GSH at pH 5.5 or 7.4, the absorbance of the mixed solution at 348 nm increased over time, indicating that Bi^3+^ and GSH coordinated to form a Bi(GS)_3_ complex and consumed GSH, which could result in the degradation of BCuS. The degradation rate of BCuS was faster under GSH/pH 5.5 conditions (Figure 3J). This accelerated degradation under acidic conditions was attributed to the additional consumption of GSH by Cu^2+^. The morphology of BCuS changed during the degradation process (Figure S14), and shape changes became more pronounced over time in the GSH/pH 5.5 group.

Based on the unique degradation behavior of BCuS, we hypothesized that the gradual release of Cu^2+^/Cu^+^ in the TME led to sustained CDT effects. To test this hypothesis, we used TMB as a probe to detect •OH generation. BCuS (50 μg mL^-1^) was incubated in a pH 5.5/H_2_O_2_/GSH system for different durations, and the change in ox-TMB absorption peaks was measured. As shown in Figures 3K and 3L, •OH generation decreased with longer incubation times. However, even after 24 hours, the generation of •OH was still detected. This finding supported the hypothesis of prolonged CDT efficacy by BCuS. Additionally, holes can also oxidize GSH. We used the DTNB colorimetric assay to quantify the consumption of GSH by BCuS under different pH values and conditions. Figures 3M and 3N show that GSH consumption was higher in the pH5.5/BCuS+US group, when compared to other conditions, indicating that holes, together with Bi^3+^ and Cu^2+^, contributed to the oxidation of GSH. We further used inductively coupled plasma optical emission spectrometry (ICP-OES) to detect the Cu^2+^ and Bi^3+^ released by BCuS at different pH values. As shown in Figure S15, BCuS at pH 5.5/H_2_O_2_/GSH released more Cu^2+^ and Bi^3+^ than at pH 7.4/H_2_O_2_/GSH, with BCuS reaching a state of continuous release. In addition, we examined the change in SDT efficiency after BCuS co-incubation with GSH and H_2_O_2_. The results showed that BCuS also had a substantial SDT performance after 12 hours of co-incubation, indicating that it could sustain the effect of SDT (Figure S16). These results showed that BCuS released Cu^2+^ in acidic TME, and used its Fenton-like reaction capability to catalyze the production of ROS from H_2_O_2_ while depleting GSH, thereby remodeling the TME (Figure 3O).

### BCuS-induced tumor cell death

Initially, we used the RhB fluorescent dye to label BCuS, to investigate the uptake of BCuS by cells. As shown in Figure S17, when BCuS-RhB was co-incubated with 4T1 cells, the cells were illuminated, and the fluorescence intensity of the cells was highest at 12 hours, indicating that after 12 hours of co-incubation, BCuS-RhB was well absorbed by cells and evenly distributed in the cytoplasm. In addition, we used a lyso-tracker green probe to determine lysosomal localization and imaging of 4T1 cells co-incubated with BCuS-RhB. As shown in Figure S18, BCuS-RhB co-localized with lysosomes with a Pearson's correlation coefficient of 0.64, indicating that BCuS entered 4T1 cells through the endocytic pathway and localized in lysosomes. This subcellular localization property was conceptually desirable because the acidic environment of lysosomes (pH 4.5-5.5) can promote BCuS dissociation and release of Cu^2+^ and Bi^3+^.

**Figure 4:**
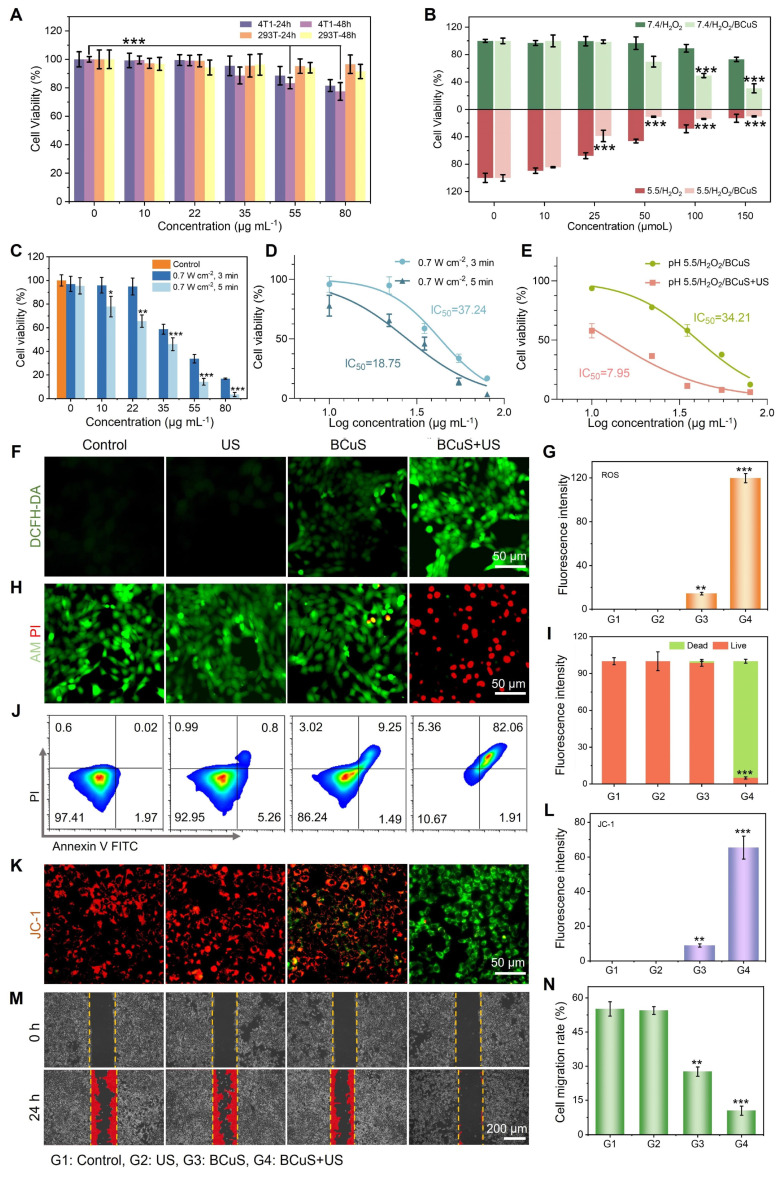
*In vitro* assessment of cell death mechanisms induced by BCuS under US treatment. (A) Relative cell viability of 4T1 and 293T cells after incubation with different concentrations of BCuS for 24 and 48 h. (B) Relative cell viability of 4T1 cells co-incubated with BCuS under different pH conditions and treated with various concentrations of H_2_O_2_. (C) Relative cell viability of 4T1 cells co-incubated with different concentrations of BCuS under different US exposure times. (D) IC_50_ of SDT at different US exposure times. (E) Comparison of IC_50_ in CDT (pH 5.5/H_2_O_2_/BCuS) alone and CDT+SDT (pH 5.5/H_2_O_2_/BCuS+US) groups. (F) DCFH-DA staining images of 4T1 cells under different treatment conditions and (G) the average fluorescence intensity analysis. (H) Calcein-AM/PI staining images of 4T1 cells under different treatment conditions and (I) the average fluorescence intensity analysis. (J) Flow cytometric analysis of 4T1 cells under different treatment conditions using Annexin-FITC and PI. (K) JC-1 staining images of 4T1 cells under different treatment conditions and (L) the average fluorescence intensity analysis. (M) Scratch assay images of 4T1 cells under different treatment conditions and (N) the relative migration rate of cells. (G1: Control, G2: US, G3: BCuS, G4: BCuS+US) Data are presented as mean ± standard deviation (n = 5) (* P < 0.05, ** P < 0.01, *** P < 0.001).

We then evaluated the SDT performance of BCuS at the cellular level and investigated its mechanisms of inducing tumor cell death and activating ICD. First, CCK-8 assays were used to assess the cytotoxicity of BCuS toward 4T1 cells and 293T cells. As shown in Figure 4A, even at high concentrations, BCuS did not significantly affect the viability of 293T cells after 24 or 48 hours of incubation. However, when 4T1 cells were incubated with BCuS for 24 or 48 hours, cell viability decreased in a dose-dependent manner, which was attributed to the long-lasting CDT effect of BCuS. To further investigate the CDT efficacy of BCuS under different pH conditions, we added varying concentrations of H_2_O_2_ to 4T1 cells at pH 7.4 and pH 5.5 (Figure 4B). The results showed that in the presence of H_2_O_2_ and under acidic conditions, the cell viability of cells incubated with BCuS was the lowest (9.7%), indicating that BCuS effectively exerted its CDT therapeutic effect in the TME.

To determine the optimal treatment time, 4T1 cells incubated with varying concentrations of BCuS were exposed to US for different durations (Figure 4C). The results showed a significant decrease in cell viability with increasing BCuS concentrations and US exposure times. Compared to the control group, cell viability dropped to 13.8% after 5 minutes of US stimulation at 0.7 W cm^-2^ and a BCuS concentration of 55 μg mL^-1^. Therefore, we selected 0.7 W cm^-2^ and 5 minutes of US exposure as the treatment conditions for subsequent experiments. Moreover, compared with SDT alone (IC_50_ = 18.75 μg mL^-1^) and CDT alone (IC_50_ = 34.21 μg mL^-1^), the lower IC_50_ value (IC_50_ = 7.95 μg mL^-1^) of CDT combined with SDT indicated that BCuS had a better therapeutic effect during US stimulation (Figures 4D and 4E).

Next, 2',7'-dichlorodihydrofluorescein diacetate (DCFH-DA) was used as a fluorescent probe to detect intracellular ROS generation by BCuS. As shown in Figures 4F and 4G, the BCuS + US group exhibited the highest fluorescence intensity, while the BCuS group showed weaker fluorescence, due to the lower amount of ROS produced through the Fenton-like reaction. Calcein-AM/propidium iodide (PI) staining was then used to detect the number of live and dead cells in each group. The results showed that the BCuS + US group had the highest number of dead cells, marked by red fluorescence (Figures 4H, 4I, and S19), validating the CCK-8 results. Flow cytometry analysis further quantitatively confirmed the effective therapeutic outcome of the BCuS + US group, with apoptosis of nearly 82% due to ROS generation (Figure 4J). This result was further confirmed by a significant up-regulation of cleaved Caspase 1 in Western blot (WB) experiments (Figure S20). JC-1 staining revealed a significant reduction in red fluorescence signals of JC-1 aggregates in the BCuS + US group (Figures 4K, 4L, and S19), indicating that ROS accumulation led to oxidative stress and mitochondrial damage, resulting in a decrease in mitochondrial membrane potential and induction of apoptosis 30. Flow cytometry analysis of JC-1 further demonstrates mitochondrial damage (Figure S21). Furthermore, apoptosis can reduce cell migration ability. The wound-healing assay showed that the migration and proliferation abilities of 4T1 cells in the BCuS + US group were significantly inhibited (Figures 4M and 4N). Thus, these results collectively indicate that BCuS generates a large amount of ROS under US stimulation, effectively inhibiting the proliferation and migration of 4T1 cells by inducing apoptosis.

**Figure 5:**
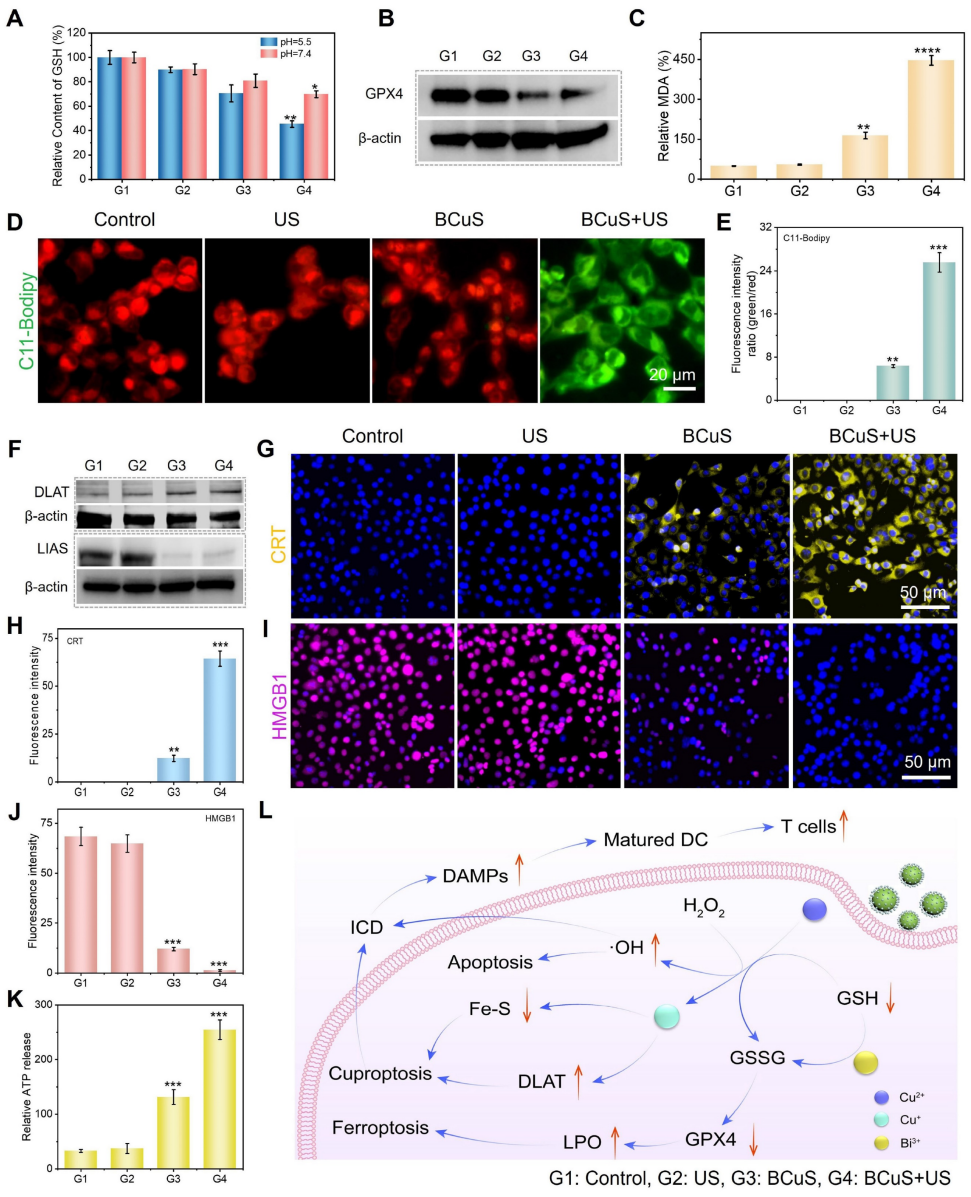
(A) Changes in GSH content in 4T1 cells. (B) WB analysis of GPX4 in 4T1 cells. (C) Evaluation of MDA levels in 4T1 cells. (D) C11-BODIPY 493/503 staining images of 4T1 cells and (E) the average fluorescence intensity analysis. (F) WB analysis of DLAT and LIAS in 4T1 cells. (G) CRT staining images of 4T1 cells and (H) the average fluorescence intensity analysis. (I) HMGB1 staining images of 4T1 cells and (J) the average fluorescence intensity analysis. (K) Relative ATP release in 4T1 cells. (L) Schematic diagram of various cell death pathways induced by BCuS in response to US stimulation and activation of robust immune responses. (G1: Control, G2: US, G3: BCuS, G4: BCuS+US) Data are presented as mean ± standard deviation (n = 5) (* P < 0.05, ** P < 0.01, *** P < 0.001).

We further investigated the consumption of intracellular GSH. As shown in Figure 5A, the BCuS + US group exhibited the highest intracellular GSH consumption under acidic conditions. GSH, as a cofactor of GPX4, has been reported to induce ferroptosis when its levels decrease 31,32. Western blot analysis showed that the BCuS + US group had the greatest reduction in GPX4 levels (Figure 5B), which was caused by increased ROS and decreased GSH content. Additionally, lipid peroxidation (LPO) is a key factor in ferroptosis. The occurrence of LPO was evaluated by assessing malondialdehyde (MDA), a major product of LPO. As shown in Figure 5C, the MDA levels in the BCuS + US group were significantly higher than in other groups, indicating the most severe LPO process. C11-BODIPY (a lipid peroxidation sensor) staining also supported these findings (Figure 5D, 5E, and S19). Thus, multiple pieces of evidence confirmed that BCuS induced ferroptosis in tumor cells during US stimulation.

Copper overload-induced cuproptosis is associated with the acetylation of enzymes in the TCA cycle, leading to protein toxicity and ultimately cell death 33. According to Tsvetkov's report, dihydrolipoamide S-acetyltransferase (DLAT) aggregation is a key marker of cuproptosis. BCuS can release Cu^2+^, so western blot analysis was performed on 4T1 cells treated with BCuS to detect DLAT levels in cells. The results showed that BCuS significantly induced DLAT aggregation, with the aggregated form increased under US stimulation, indicating that SDT enhanced cuproptosis (Figure 5F). As expected, WB analysis further confirmed that BCuS downregulated the expression of lipoic acid synthase (LIAS), which plays a crucial role in inducing cuproptosis (Figure 5F). Therefore, SDT in the TME induced the release of Cu^2+^ and Bi^3+^ from BCuS, leading to massive ROS generation and intracellular GSH depletion, which synergistically caused cuproptosis and ferroptosis, ultimately inducing tumor cell death through multiple pathways.

Some studies have reported that cuproptosis or ROS stimulation activates ICD, thereby triggering systemic anti-tumor immunity 34,35. The main hallmarks of ICD include CRT exposure and the secretion of HMGB1 and ATP 35. To determine whether cuproptosis and sustained intracellular ROS generation induced ICD, we evaluated CRT expression on the cell membrane and the release of HMGB1 in all groups using immunofluorescence staining. As shown in Figures 4G, 4H, and S19, CRT was significantly exposed on the cell membrane surface of 4T1 cells in the BCuS+US group. This externalization acted as an “eat-me” signal, promoting the migration and maturation of dendritic cells and inducing an anti-tumor immune response. Moreover, Figures 4I, 4J, and S19 show that HMGB1 was successfully released in the BCuS+US group. HMGB1 stabilized nucleosome structures and regulated gene expression, thereby activating relevant signaling pathways to initiate immune responses. Finally, ATP secretion was monitored using an ATP assay kit (Figure 4K). The extensive release of extracellular ATP in the BCuS + US group was consistent with the observed CRT externalization and HMGB1 release. These results collectively indicated that during US stimulation, BCuS enhanced tumor cell cuproptosis and ferroptosis through sustained ROS generation, GSH depletion, and Cu^2+^ release, effectively inducing ICD and activating the immune response (Figure 4L).

### Evaluation of the therapeutic efficacy of BCuS *in vivo*

**Figure 6:**
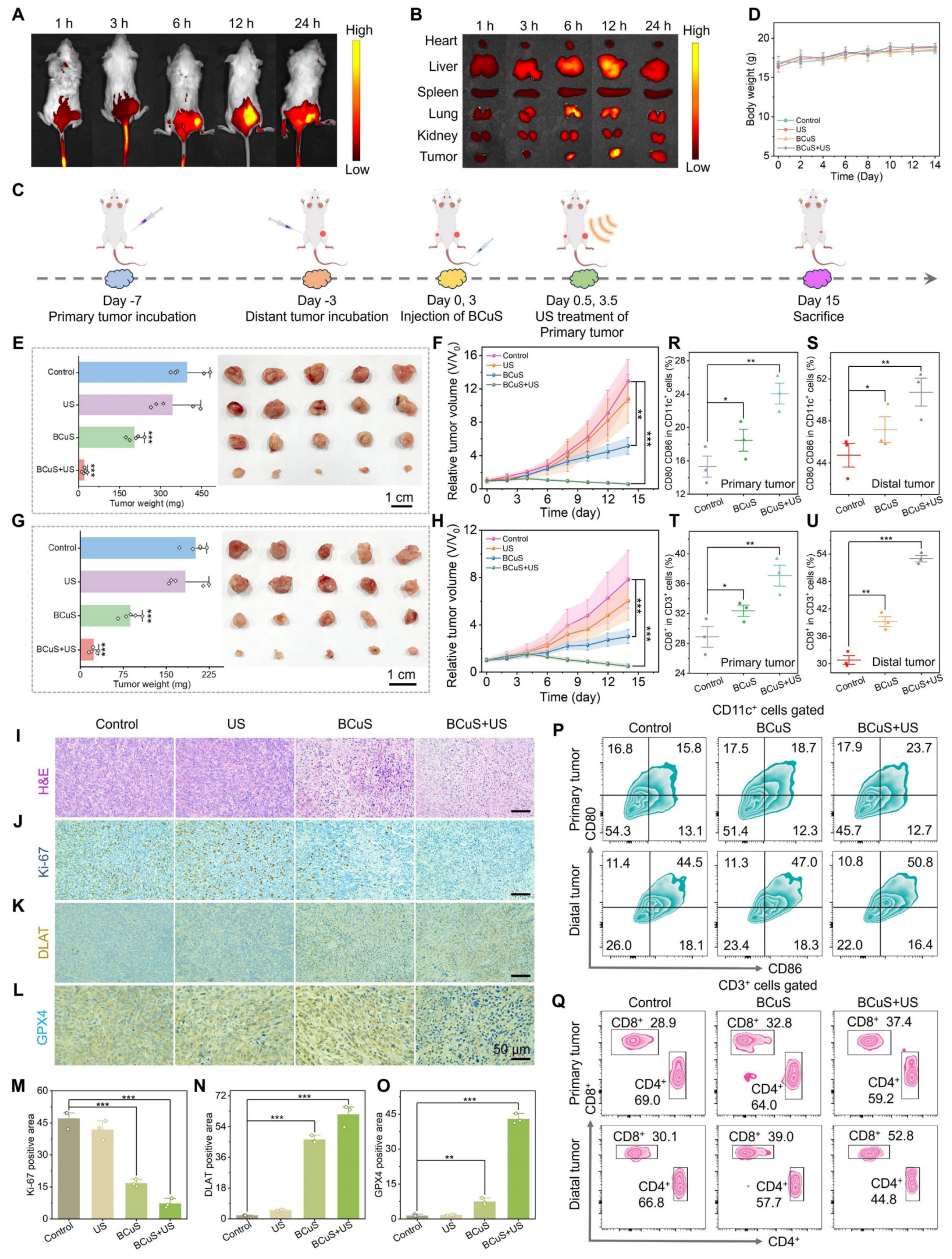
*In vivo* evaluation of the antitumor efficacy of BCuS. Accumulation of BCuS-IR780 at (A) tumors and (B) organs at different time points after intravenous injection. (C) Schematic diagram of the treatment regimen for mice. (D) Body weights of mice in different groups during the treatment period. After 14 days of treatment, (E) average tumor weight and images of primary tumors and (F) average growth curves of primary tumors in different groups. After 14 days of treatment, (G) average tumor weight and images of distant tumors and (H) average growth curves of distant tumors in different groups. (I) H&E staining images of primary tumor tissue sections. Primary tumor tissue sections (J) Ki-67 staining, (K) DLAT staining, and (L) GPX4 staining images. Relative quantitative analysis of (M), (N), and (O) for Figures (J), (K), and (L), respectively. Flow cytometric analysis of (P) mature DCs and (Q) T lymphocytes in primary and distant tumors. Relative quantitative analysis of mature DCs (R) in primary tumors and (S) in distant tumors. Relative quantitative analysis of T lymphocytes (T) in primary tumors and (U) in distant tumors. Data are presented as mean ± standard deviation (n = 5) (* P < 0.05, ** P < 0.01, *** P < 0.001).

Based on the properties of BCuS and its promising therapeutic effects against tumor cells, we evaluated its therapeutic efficacy in a mouse tumor model. First, we measured the accumulation of BCuS in tumors and major organs using *in vivo* fluorescence imaging. To facilitate this imaging, the near-infrared fluorescent dye IR780 was modified on the surface of BCuS, forming the luminescent BCuS-IR780 nanocomposite. As shown in Figure 6A, the fluorescence intensity in the tumor reached its peak 12 hours after intravenous injection, then gradually decreased. Similarly, the fluorescence intensity in various organs followed a similar trend (Figure 6B), indicating that BCuS-IR780 effectively accumulated at the tumor site and exhibited good metabolic clearance. Based on these results, we selected 12 hours post-injection as the optimal time for subsequent treatments in mice.

To evaluate the antitumor efficacy of BCuS *in vivo*, mice were randomly divided into four groups: (1) control, (2) US, (3) BCuS, and (4) BCuS + US groups. The treatment protocol is illustrated in Figure 6C. Mice in the BCuS and BCuS+US groups received intravenous injections of BCuS, followed by SDT treatment in the BCuS+US group 12 hours later. During the 14-day treatment period, the body weight of all groups remained relatively stable, indicating that BCuS did not adversely affect this parameter (Figure 6D). Compared with the control and US groups, tumor inhibition in the BCuS group was 48.0%, while the BCuS + US group exhibited significant growth inhibition of 92.3% (Figures 6E, 6F, and S22). These results were consistent with *in vitro* cell experiments, indicating that BCuS effectively suppressed tumor proliferation. Notably, compared to the control and US groups, the BCuS and BCuS+US groups also showed significant inhibition of tumor growth at distant sites (Figures 6G, 6H, and S23). Specifically, distant tumor inhibition was 43.6% for the BCuS group and as high as 87.3% for the BCuS+US group, indicating a significant therapeutic effect and suggesting that ICD activation in the primary tumor triggered a systemic immune response.

Next, we performed immunohistochemical analysis on tumor tissues. Hematoxylin and eosin (H&E) staining of tumor sections showed severe cell damage and significant morphological loss in the BCuS+US group, indicating pronounced apoptosis (Figure 6I). Furthermore, the BCuS+US group had the lowest level of Ki-67 positive staining, indicating the most effective inhibition of tumor cell proliferation (Figures 6J and 6M). These results agreed with the tumor growth curve and confirmed that BCuS effectively suppressed tumor growth during US stimulation. Immunohistochemical analysis further supported the occurrence of cuproptosis and ferroptosis. Figures 6K and 6N show positive brown staining in the BCuS group and the combination group, with a broader distribution of brown in the BCuS+US group, indicating that BCuS promoted DLAT aggregation, confirming the occurrence of cuproptosis. In addition, GPX4 immunohistochemical staining showed significant downregulation of GPX4 expression in the BCuS+US group, suggesting the occurrence of ferroptosis (Figures 6L and 6O).

We then performed flow cytometry to quantify the proportion of mature DCs and activated T lymphocytes in tumor tissues after treatment. Co-expression of CD80 and CD86 is a hallmark of DC maturation. As shown in Figures 6P, 6R, and 6S, BCuS+US treatment effectively promoted DC maturation in lymph nodes. Compared to the control group, the proportions of DCs in the primary and distant tumors of the BCuS+US group increased by 1.5 and 1.1 fold, respectively. DC maturation can regulate T cell proliferation, thereby mediating downstream immune responses. In these responses, CD8^+^ T cells play a critical role in antitumor immunotherapy and are crucial for regulating adaptive immunity. Therefore, we further assessed the number of CD8^+^ T cells. As shown in Figures 6Q, 6T, and 6U, the proportions of CD8^+^ T cells in primary and distant tumors increased from 28.9% and 30.1% in the control group to 37.4% and 52.8%, respectively, after BCuS+US treatment. The gating strategy is shown in Figures S24 and S25. These data collectively suggested that BCuS activated systemic immunity during US stimulation, significantly inhibiting the proliferation of distant untreated tumors.

We also evaluated the biocompatibility and toxicity of BCuS using hemolysis tests, blood routine analysis, and H&E staining of tissues. The hemolysis test showed that the hemolysis was below the safety threshold (5%) defined by ISO/TR 7406 within the administered dosage range (Figure S26). After 14 days of intravenous injection of BCuS, H&E staining of various organs and blood routine indicators showed no significant differences, when compared with the control group, indicating the excellent biocompatibility and biosafety of BCuS (Figures S27 and S28).

### ICD reverses the immunosuppressive characteristics of the TME

**Figure 7:**
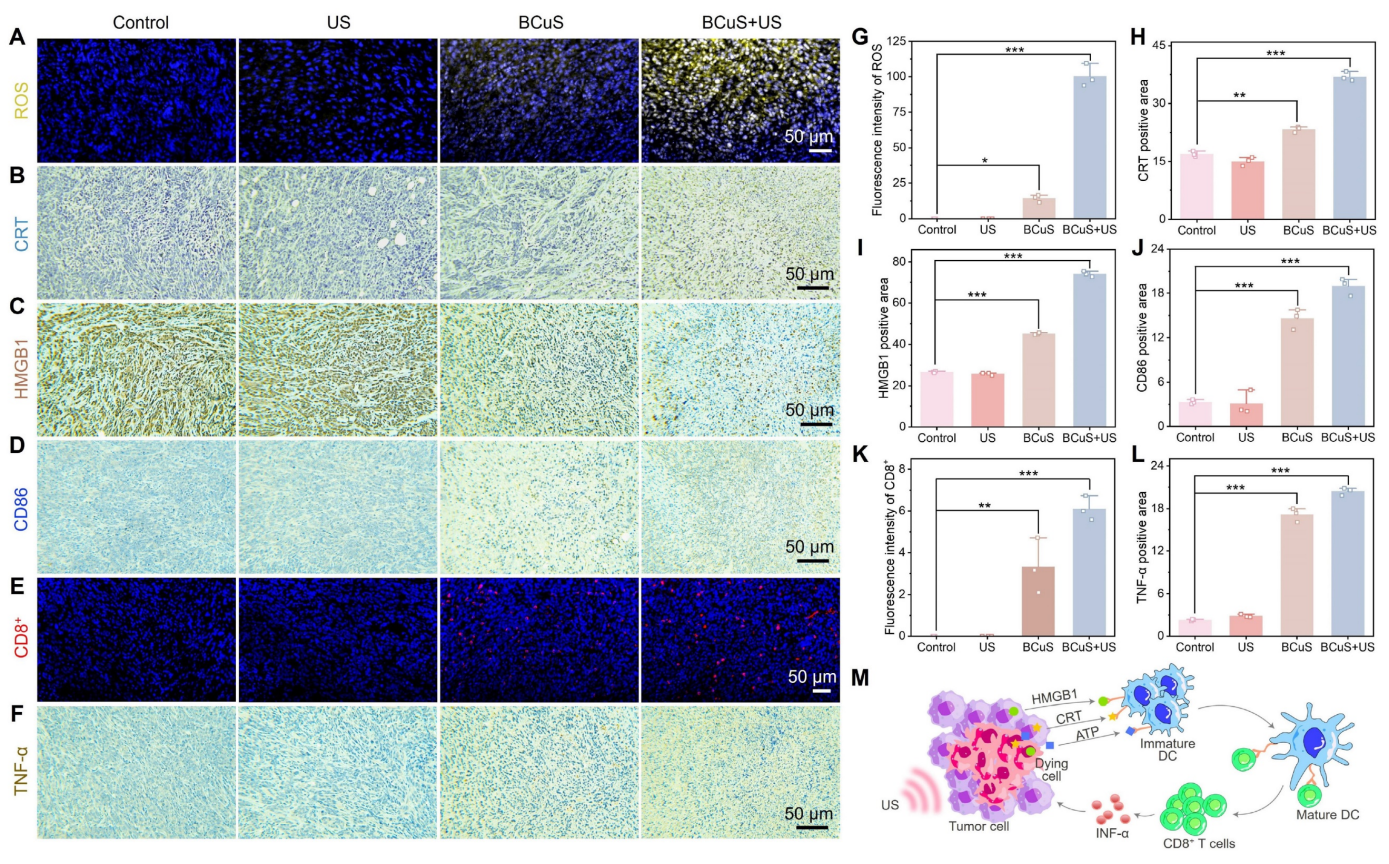
ICD reverses TME immune suppression. (A) ROS immunofluorescence staining in primary tumor tissue. (B) CRT, (C) HMGB1, and (D) CD86 immunohistochemical staining in primary tumor tissue. (E) CD8^+^ immunofluorescence staining in primary tumor tissue. (F) TNF-α immunohistochemical staining in primary tumor tissue. Relative quantification of (G) ROS, (H) CRT, (I) HMGB1, (J) CD86, (K) CD8^+^, and (L) TNF-α. (M) Schematic diagram demonstrating how BCuS activates the tumor immune system. Data are presented as mean ± standard deviation (n = 3) (* P < 0.05, ** P < 0.01, *** P < 0.001).

Based on flow cytometry results, we proposed that cuproptosis and extensive ROS generation led to ICD, effectively reversing the immunosuppressive characteristics of the TME. This conclusion was further supported by immunofluorescence and immunohistochemical analyses. As shown in Figures 7A and 7G, the BCuS + US group exhibited the highest fluorescence intensity, indicating significant ROS production within the tumor, and confirming that BCuS generated a large amount of ROS during US and H_2_O_2_ stimulations. Immunohistochemical results further showed that during US stimulation, BCuS induced widespread exposure of CRT in tumor tissues and significant translocation of HMGB1 from the nucleus to the cytoplasm (Figures 7B, 7C, 7H, and 7I), confirming that ICD was effectively triggered *in vivo*. Moreover, Figures 7D, 7E, 7J, and 7K show that the combination group had the most extensive infiltration of DCs and cytotoxic CD8^+^ T cells at the tumor site. This suggested strong recruitment of immune cells into the tumor, activating the immune system and enhancing therapeutic efficacy. At the same time, the secretion of pro-inflammatory cytokine TNF-α in the BCuS + US group was significantly increased, when compared with the control group (Figures 7F and 7L). Collectively, these results showed that US-activated BCuS-induced ICD reversed the immunosuppressive characteristics of the TME, and further activated systemic immunity, effectively inhibiting the growth of distant tumors while eliminating primary tumors (Figure 7M).

### Anti-metastatic effect and therapeutic mechanism of BCuS under US stimulation

**Figure 8:**
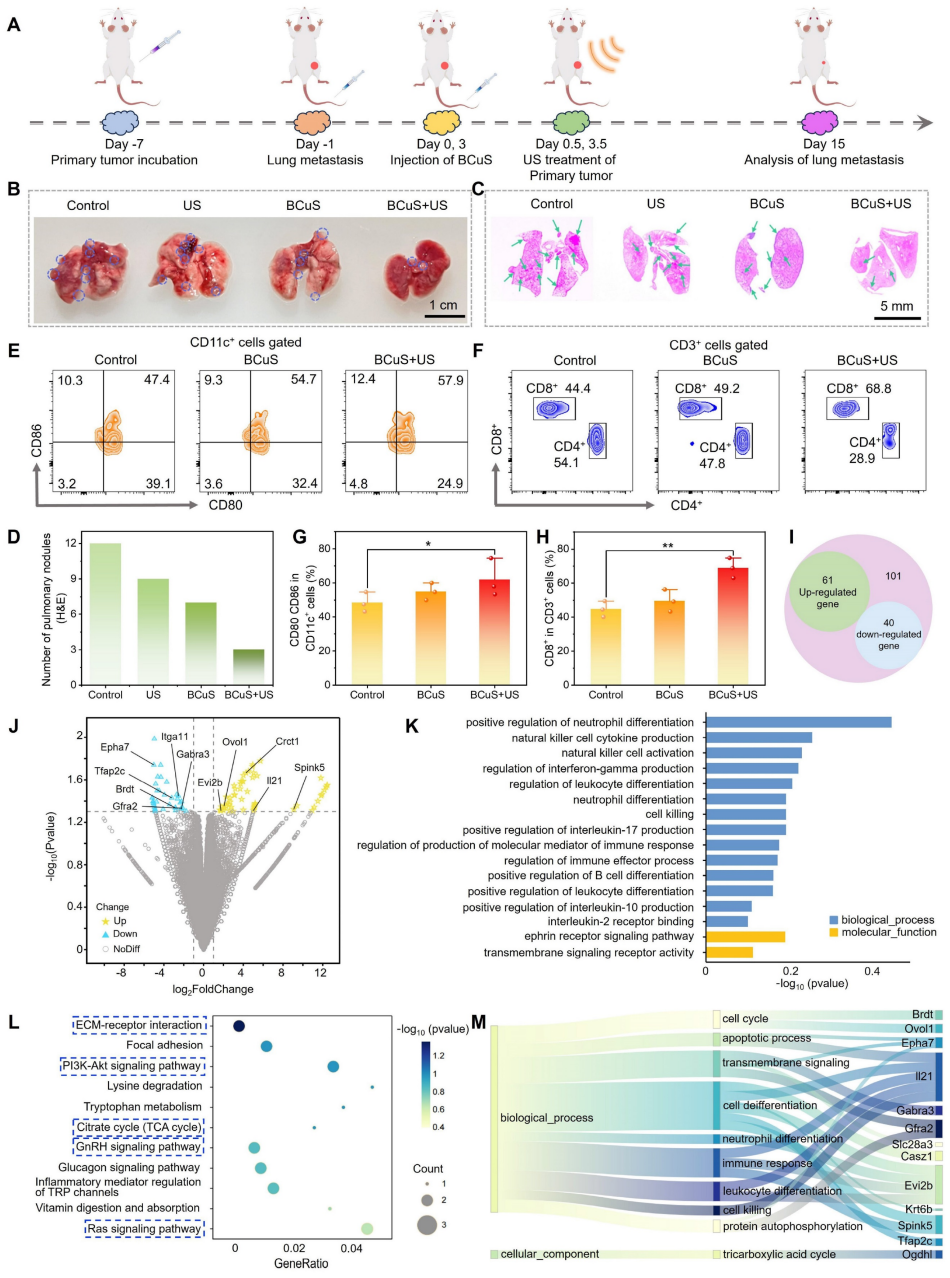
*In vivo* antimetastatic effects and mechanistic insights revealed by RNA sequencing. (A) Establishment of the lung metastasis model and the treatment regimen. (B) Images of visible lung tumor nodules. (C) H&E staining of the lungs. (D) Number of pulmonary nodules in H&E staining. Flow cytometry analysis of (E) mature DCs and (F) CD8^+^ T cells in the spleen. Relative quantification of (G) mature DCs and (H) CD8^+^ T cells. (I) Venn diagram of differentially expressed genes (DEGs) between the control and combination treatment groups from RNA sequencing. (J) Volcano plot of DEGs. (K) GO enrichment analysis of differentially regulated biological processes post-treatment. (L) KEGG pathway enrichment analysis bubble plot post-treatment. (M) Sankey diagram illustrating genes associated with cell death, immunity, and transmembrane signaling. Data are presented as mean ± standard deviation (n = 3) (* P < 0.05, ** P < 0.01, *** P < 0.001).

Based on flow cytometry data and the observed inhibition of distant tumor growth in the combination group, we further investigated the potential of BCuS to enhance systemic immune activation through cuproptosis and ROS-mediated ICD during sonocatalytic conditions. As shown in Figure 8A, we established a mouse lung metastasis model to test treatment efficacies. Lung tissues were collected and analyzed 15 days after the primary tumor treatment. In Figure 8B, a large number of metastatic nodules were observed on the surface of lung tissues in the control group, whereas the number of nodules in the combination group was minimal. Consistent with the digital images, H&E staining of the lungs confirmed that BCuS effectively inhibited lung metastasis during US irradiation (Figures 8C and 8D). Under the effects of ICD, tumor cells underwent death and released DAMPs, which were recognized by immature DCs, leading to their maturation and the activation of T lymphocytes, thereby initiating a systemic antitumor immune response. To evaluate this process, we analyzed the maturation of DCs in the spleen. As shown in Figures 8E and 8G, the proportion of mature DCs in the spleen was 57.9% in the combination group, 10.5% higher than that in the control group. In addition, we assessed CD8^+^ T cells, one of the most cytotoxic T lymphocytes that directly determine tumor-killing capacity. Figures 8F and 8H show that the activation of CD8^+^ T cells in the spleens of the BCuS+US group was 68.8%, which was 1.55 times higher than that in the control group, indicating that the combination group had the most significant effect on inhibiting tumor growth and metastasis. Together, these results showed that BCuS-induced cuproptosis and ROS generated during US stimulation-mediated ICD activated systemic immune responses and enhanced long-term resistance to tumor metastasis.

To identify the cell death mechanisms mediated by BCuS during US stimulation, we performed transcriptome sequencing of primary tumors from treated and untreated mice to identify differentially expressed genes. Compared to the control group, the BCuS + US group showed differential expression of 101 genes, with 61 genes upregulated and 40 genes downregulated (fold change ≥ 1.5, p < 0.05) (Figure 8I). The heatmap further illustrated the significant differences in gene expressions between the combination and control groups (Figure S29). The volcano plot displayed the expression of these transcripts (Figure 8J). Among the downregulated genes, *Gfra2, Spink5, Crct1,* and *Ovol1* were associated with inhibiting tumor cell proliferation, migration, and infiltration, as well as promoting tumor cell apoptosis 36-39. In addition, the abnormal expression of genes such as *Itga11, Brdt*, and *GABR*_A3_ further confirmed the occurrence of apoptosis 40-42. IL-21, mainly produced by activated CD4^+^ T cells, played a critical role in enhancing the effector function of CD8^+^ T cells 43. Upregulation of IL-21 indicated its activation, promoting the development of CD4^+^ T cells and expanding the CD8^+^ T cell population, demonstrating the initiation of an immune response. Moreover, the abnormal expression of genes such as *Epha7* and *Evi2b* also supported the occurrence of an immune response 44,45. Additionally, the downregulation of TFAP2C blocked the transition of the cell cycle from the G1 to S1, decreased GPX4 levels, and increased MDA, ROS, and Fe^2+^ levels, thereby inducing ferroptosis 46. Therefore, the downregulation of TFAP2C confirmed the occurrence of ferroptosis. Notably, genes related to cuproptosis also showed abnormal expressions. For example, the downregulation of *KRT6B* increased sensitivity to copper ion carriers, thereby leading to cuproptosis 47. These results indicated that BCuS induced multiple forms of cell death during US stimulation, effectively inhibiting tumor growth and activating immune responses. This comprehensive mechanism involved both direct tumor-killing effects and the activation of systemic immune responses.

To identify biological pathways associated with differentially expressed genes, we performed Gene Ontology (GO) and Kyoto Encyclopedia of Genes and Genomes (KEGG) pathway enrichment analyses. As expected, GO enrichment analyses highlighted pathways related to cell death, immune responses, leukocyte and B cell differentiation, and transmembrane receptor signaling processes (Figure 8K). KEGG pathway analyses showed that the PI3K-Akt, GnRH, and Ras signaling pathways, as well as the TCA cycle and extracellular matrix-receptor interaction, were significantly enriched after treatment. These pathways were closely related to the products generated by BCuS during US stimulation (Figures 8L and 8M). Therefore, our findings at the genetic level confirmed that BCuS induced multiple forms of cell death and activated systemic immunity during SDT, providing an effective combined therapeutic strategy for tumor treatment.

## Conclusion

In summary, BCuS was capable of eliciting a potent immune response, achieving excellent tumor treatment outcomes, and preventing tumor recurrence. The successful reversal of the TME immune suppression by BCuS was partly attributed to its ability to undergo a Fenton-like reaction in the acidic TME, facilitating the conversion of Cu^2+^ to Cu^+^ and the generation of substantial amounts of ROS. This, in turn, promoted cuproptosis and ROS-induced ICD. Additionally, BCuS leveraged Cu^2+^, Bi^3+^, and holes to deplete GSH, disrupting the unique antioxidant defenses of tumor cells and inducing both apoptosis and ferroptosis. These mechanisms collectively enhanced DC maturation, increased T lymphocyte recruitment and infiltration, stimulated systemic immune responses, and led to the suppression of both primary and distant tumors, as well as a reduction in lung metastasis. Additionally, the analysis of BCuS's SDT performance showed that its p-n heterojunction characteristics facilitated the rapid separation of electron-hole pairs while inhibiting their recombination. This led to the generation of significant amounts of ROS during US stimulation, resulting in oxidative stress in tumors. Both cellular and *in vivo* anti-tumor studies further substantiated the ability of BCuS to inhibit tumor growth and metastasis during US treatment. In summary, BCuS, as a p-n heterojunction-based sonosensitizer, showed excellent anti-tumor effects with favorable biocompatibility and degradability, providing a unique perspective for activating robust immune responses.

## Supplementary Material

Supplementary experimental section and figures.
