# Supplementary material for: A p-n heterojunction sonosensitizer for improved sono-immunotherapy via induction of multimodal cell death mechanisms
Source: Theranostics. 2025 Jan 27;15(7):2737–56. doi: 10.7150/thno.106999 (PMC11898295; doi:10.7150/thno.106999)
Supplement: Supplementary file 1 — Supplementary experimental section and figures. [file thnov15p2737s1.pdf]

A p-n heterojunction sonosensitizer for improved sono-immunotherapy via induction of multimodal cell death mechanisms

Sijia Wu[1,2], *Qian Wang*[2], *Jun Du*[2], *Qingxuan Meng*[2], *Yuhao Li*[2,*], *Yuqing Miao*[2], *Qing Miao*[1,*], *Jingxiang Wu*[1,*]

1. Department of Anesthesiology, Shanghai Chest Hospital, Shanghai Jiao Tong University, School of Medicine, Shanghai 200030, China.
2. School of Materials and Chemistry, Institute of Bismuth Science, Shanghai Collaborative Innovation Center of Energy Therapy for Tumors, University of Shanghai for Science and Technology, Shanghai 200093, China

Corresponding authors:

Yuhao Li: E-mail yhli@usst.edu.cn

Qing Miao: E-mail miaoqmz@163.com

Jingxiang Wu: E-mail wu_jingxiang@sjtu.edu.cn

Experimental section

Characterization

The morphology and crystalline structure of the samples were examined using transmission electron microscopy (TEM, Hitachi HT7800, Japan) and high-resolution TEM (FEI Tecnai G2 F20, USA). Phase composition analysis was performed using an X-ray powder diffractometer (XRD, Bruker D8 advance, Germany) to obtain X-ray diffraction patterns. The chemical states of the elements were analyzed using X-ray photoelectron spectroscopy (XPS, Thermo K-Alpha, USA). Absorption spectra were acquired using a spectrophotometer (Shimadzu UV-1900, Japan) to evaluate the absorption characteristics of the samples. Fluorescence spectra were acquired using a fluorescence spectrophotometer (FS5, Edinburgh, UK). Zeta potential and dynamic light scattering (DLS) measurements were conducted using a nanoparticle size analyzer (Malvern Zetasizer lab, England). Fourier-transform infrared (FT-IR) spectra were obtained using an infrared spectrometer (Shimadzu IRTracer-100, Japan) for molecular structure and chemical composition analysis. The content of reactive oxygen species (ROS) and oxygen vacancies in the samples was measured using an electron spin resonance (ESR) spectrometer (Bruker EMXplus, Germany). The content of Cu^{2+} and Bi^{3+} was quantified using inductively coupled plasma optical emission spectrometry (ICP-OES, Thermo Fisher iCAP 7200, USA).

EPR detection of ROS

BCuS (50 μg mL^{-1}) was dissolved in a solution of 2,2,6,6-tetramethylpiperidine-1-oxyl (TEMPO, ≥ 98%, Adamas, China) and 5,5-dimethyl-1-pyrroline N-oxide (DMPO, 99.9%, Adamas, China), and then subjected to US treatment (1 W cm^{-2}) for 1 min. Characteristic peaks were obtained using an EPR spectrometer to assess the generation of $\bullet O_2^-$ and $^1O_2$. To further verify the chemical kinetics of BCuS, BCuS (50 μg mL^{-1}), H_2O_2 (0.5 mM), GSH (10 mM), and DMPO were dissolved in PBS (pH = 5.5) and incubated at room temperature for 10 min. EPR signals were then captured to analyze the chemodynamic process.

Degradation behavior of BCuS

BCuS (100 μg mL^{-1}) and GSH (10 mM) were mixed in PBS at different pH values (7.4 or 5.5) and incubated in an orbital shaker at 37 °C and stirred at 250 rpm. Subsequently, the degradation behavior of BCuS was observed at various time points using TEM. Finally, the absorbance of the degradation system at 348 nm was monitored using a spectrophotometer at different time intervals.

Quantified the contents of Cu^{2+} and Bi^{3+}

BCuS (100 μg mL^{-1}), GSH (10 mM), and H_2O_2 (0.5 mM) were mixed in PBS at different pH values (7.4 or 5.5) and incubated in a 37 °C orbital shaker. The mixed solution is then centrifuged at a specific time, the supernatant solution is removed and digested with a nitric acid solution at high temperatures. Finally, the solution was collected, and the contents of Cu^{2+} and Bi^{3+} were quantified by the ICP-OES method.

Photoelectrochemical performance evaluation

The photoelectric current, electrochemical impedance, and Mott–Schottky curves of CuS, $Bi_2O_{3-x}S_x$, and BCuS were measured using an electrochemical workstation (CHI 660E, China). CuS, $Bi_2O_{3-x}S_x$, and BCuS were first deposited onto fluorine-doped tin oxide (FTO) glass to form working electrodes. These electrodes were then placed in a quartz cell, where they were part of a standard three-electrode system comprising an Ag/AgCl reference electrode and a platinum foil counter electrode. The standard three-electrode system was immersed in PBS solution (0.1 M, pH 7.4). Photoelectric current curves were recorded under continuous illumination with a 300 W Xenon lamp (100 mW cm^{-2}) as well as in the dark. Electrochemical impedance measurements were conducted in a 50 mL mixed solution containing 1 mM $K_3[Fe(CN)_6]$, 1 mM $K_4[Fe(CN)_6]$, and 0.5 M KCl. The Mott–Schottky curves for CuS, $Bi_2O_{3-x}S_x$, and BCuS were obtained in a 50 mL $NaSO_4$ solution (0.5 M) within a frequency of 1000 Hz and a voltage range of 2 to −1 V.

Cellular uptake

Rhodamine B (0.2 mg) and 1 mg of BCuS were evenly dispersed in pure water and stirred in the dark for 24 hours. Then, the BCuS was washed 3 times with PBS to obtain Rhodamine B-labeled BCuS (BCuS-RhB). Subsequently, we evenly distributed the 4T1 cells in confocal dishes. After 24 hours, BCuS-RhB (55 μg mL^{-1}) dispersed in medium RPMI-1640 was added to the confocal dish. Then, at 2, 4, 6, 8, and 12 hours, photographs of cell uptake were taken with a laser scanning confocal microscope (CLSM, Zeiss LSM 800, Germany).

Lysosomal colocalization

We evenly cultured the 4T1 cells in confocal dishes. After 24 hours, BCuS-RhB (55 μg mL^{-1}) dispersed in medium 1640 was added. Then, at 8 hours, lysosomal localization staining was performed using Lyso-Tracker Green (Beyotime Biotechnology, China) for 30 minutes. Finally, the localization of BCuS-RhB was observed and photographed by CLSM.

Assays for intracellular ROS production

Intracellular ROS production was detected using a reactive oxygen species detection kit (DCFH-DA fluorescent probe, Beyotime Biotechnology, China). 4T1 cells were seeded into 24-well plates at a density of 5×10^4 cells per well and incubated for 24 h. BCuS (55 μg mL^{-1}) was then dispersed in RPMI-1640 and added to groups (3) and (4), which were further incubated for 24 h. Subsequently, cells in groups (2) and (4) were subjected to US stimulation and then incubated for an additional 6 h. DCFH-DA was added according to the manufacturer's instructions, and the cells were incubated in the dark for 30 min. Finally, cells were washed twice with PBS and observed using an inverted fluorescence microscope (Olympus IX73, Japan) to visualize the staining results (DCFH-DA: green).

Live/dead cell staining

Live and dead cells were indicated using Calcein-AM and PI cell staining probes (Beyotime Biotechnology, China). 4T1 cells were seeded into 24-well plates at a density of 5×10^4 cells per well and incubated for 24 h. BCuS (55 μg mL^{-1}) was then dispersed in RPMI-1640 and added to groups (3) and (4), which were further incubated for 24 h. Cells in groups (2) and (4) were subjected to US stimulation and then incubated for an additional 6 h. Calcein-AM and PI were added according to the manufacturer's instructions, and the cells were incubated in the dark for 30 min. Finally, cells were washed twice with PBS and observed using an inverted fluorescence microscope to visualize the staining results (Calcein-AM: green, PI: red).

Mitochondrial membrane potential assay

Mitochondrial membrane potential changes were detected using a mitochondrial membrane potential assay kit (Beyotime Biotechnology, China). JC-1 staining solution and wash solution were prepared according to the kit instructions. 4T1 cells were seeded into 24-well plates at a density of 5×10^4 cells per well and incubated for 24 h. BCuS (55 μg mL^{-1}) was then dispersed in RPMI-1640 and added to groups (3) and (4), which were further incubated for 24 h. Cells in groups (2) and (4) were subjected to US stimulation and then incubated for an additional 6 h. JC-1 staining solution was added according to the manufacturer's instructions, and the cells were incubated in the dark for 20 min. Finally, cells were washed with the wash solution and observed using an inverted fluorescence microscope to visualize the staining results.

Cell scratch assay

4T1 cells were seeded into 6-well plates at a density of 1×10^5 cells per well and incubated for 24 h. A scratch was made in each well, and images were taken. After 0 h, BCuS (55 μg mL^{-1}) was dispersed in RPMI-1640 and added to groups (3) and (4), which were further incubated for 12 h. Cells in groups (2) and (4) were then subjected to US stimulation and incubated for an additional 12 h. Subsequently, cells were washed

twice with PBS, and images of the scratch areas were taken. Cell migration rates were calculated using the following formula: Migration rate = (The area of the cell gap at 0 h − The area of the cell gap at 24 h)/The area of the cell gap at 0 h.

Flow cytometry analysis of JC-1

Four groups (control, US, BCuS, BCuS + US) cells were treated differently, and then suspension cells and adherent cells were collected for each group. We added a certain amount of prepared JC-1 staining solution to the collected cells, mixed well, and incubated at 37 °C for 20 minutes. We added a certain amount of prepared JC-1 staining solution to the collected cells, mixed well, and incubated at 37 °C for 20 minutes. The cells were then washed twice with the prepared JC-1 staining buffer and the supernatant was collected. Finally, the analysis was performed by flow cytometry.

Intracellular GSH consumption

Intracellular GSH levels were measured using a total glutathione detection kit with DTNB. 4T1 cells were seeded into 6-well plates at a density of 1×10^5 cells per well and incubated for 24 h. BCuS (55 μg mL^{-1}) was then dispersed in RPMI-1640 and added to groups (3) and (4), which were further incubated for 24 h. Cells in groups (2) and (4) were subjected to US stimulation and incubated for an additional 12 h. Cells were washed twice with PBS, trypsinized, and centrifuged to remove the supernatant. The cell pellet was resuspended in three times its volume of protein extraction reagent. After thorough vortexing, the sample underwent three rapid freeze-thaw cycles between liquid nitrogen and a 37 °C water bath. The sample was then incubated at 4 °C for 5 min. Finally, the supernatant was collected by centrifugation and mixed with DTNB solution according to the manufacturer’s instructions to assess GSH content.

Detection of malondialdehyde

Malondialdehyde (MDA) levels were measured using an MDA detection kit (Beyotime, China) to evaluate lipid peroxidation (LPO) levels. 4T1 cells were seeded

into 24-well plates at a density of 5×10^4 cells per well and incubated for 24 h. BCuS (55 μg mL^{-1}) was then dispersed in RPMI-1640 and added to groups (3) and (4), which were further incubated for 24 h. Cells in groups (2) and (4) were subjected to US stimulation and incubated for an additional 12 h. Cells were then collected and lysed on ice, and the supernatant was obtained by centrifugation. The supernatant was mixed with MDA detection reagent according to the manufacturer's instructions and heated at 100 °C for 20 min. After cooling to room temperature, the sample was centrifuged, and the absorbance was measured at 532 nm.

Detection of intracellular lipid droplets

Intracellular lipid droplets were detected using a lipid droplet green fluorescence detection kit (BODIPY, Beyotime, China). 4T1 cells were seeded into 6-well plates at a density of 1×10^5 cells per well and incubated for 24 h. BCuS (55 μg mL^{-1}) was then dispersed in RPMI-1640 and added to groups (3) and (4), which were further incubated for 24 h. Cells in groups (2) and (4) were subjected to US stimulation and incubated for an additional 12 h. Cells were then washed twice with PBS, stained with the staining solution according to the manufacturer's instructions, and incubated in the dark at 37 °C for 15 min. Finally, cells were washed twice with PBS and observed using an inverted fluorescence microscope to visualize the staining results (green fluorescence).

In vivo fluorescence imaging

IR-780, a near-infrared fluorescence dye (1.2 mg), was mixed with BCuS (0.5 mg mL^{-1}, 2 mL) in dimethyl sulfoxide (DMSO, 99.9%, Adamas, China) and stirred in the dark for 24 h. After centrifugation and washing, the BCuS-IR780 composite was resuspended in PBS. BCuS-IR780 (1 mg mL^{-1}, 100 μL) was administered to the mice via tail vein injection. Fluorescence images were captured at different time points (1, 3, 6, 12, and 24 h) using an animal imaging system (PerkinElmer IVIS Lumina III, USA) with excitation at λ_{ex} = 630 nm and emission at λ_{em} = 800 nm. Finally, the mice were euthanized, and their major organs and tumors were collected for fluorescence imaging.

Hemolysis assay

Blood was collected from the mouse's eyeball and placed in an anticoagulant tube, then mixed with PBS (pH = 7.4) before centrifugation. The supernatant was discarded, and the washing step was repeated three to four times. The red blood cells were resuspended in a specific volume of PBS. This suspension was incubated with deionized water, PBS, and varying concentrations of BCuS (5, 10, 20, 35, 55, and 80 μg mL^{-1}) for 6 h. Deionized water served as a positive control, while PBS acted as a negative control. After centrifugation, the supernatant was transferred to a 96-well plate for absorbance measurement at 570 nm. The hemolysis rate was calculated using the following formula: Hemolysis rate (%) = $(A_{sample} - A_{PBS})/(A_{water} - A_{PBS}) \times 100\%$, A_{sample} represents the absorbance of the red blood cell suspension mixed with different concentrations of BCuS.

Biosafety and organ damage analysis

BCuS (1 mg mL^{-1}, 100 μL) and PBS (100 μL, control) were administered to BALB/c mice via tail vein injection, and the mice were monitored for 14 days. Blood samples were subsequently collected from each mouse for various blood parameter analyses. Following this, the mice were euthanized, and their hearts, livers, spleens, lungs, and kidneys were harvested. The organs were fixed in formalin for subsequent sectioning and Hematoxylin and Eosin (H&E) staining.

Flow cytometry analysis of tumor immune microenvironment

Mice from groups (1), (3), and (4) were euthanized, and their primary tumors, distal tumors, and spleens were harvested and homogenized. Tumor tissues were processed using an immune cell tumor dissociation kit, while spleen tissues were lysed with red blood cell lysis solution. Samples were then treated with PBS containing anti-CD16/CD32 antibodies to block Fc receptors. Following this, immune cells were stained with antibodies against CD45, CD3, CD4, CD8, CD11c, CD80, and CD86 and incubated for 30 min. After stopping the staining with PBS, cells were further stained

and fixed using the Zombie Violet Fixable Viability Kit. Finally, flow cytometry was performed to analyze the samples.

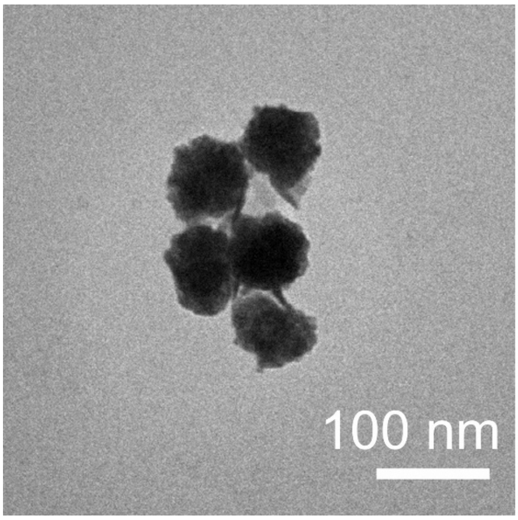

Figure S1. TEM image of $Bi_2O_{3-x}S_x$.

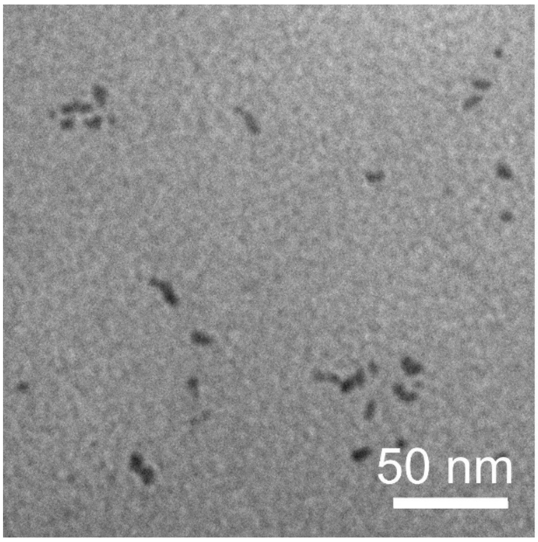

Figure S2. TEM image of CuS.

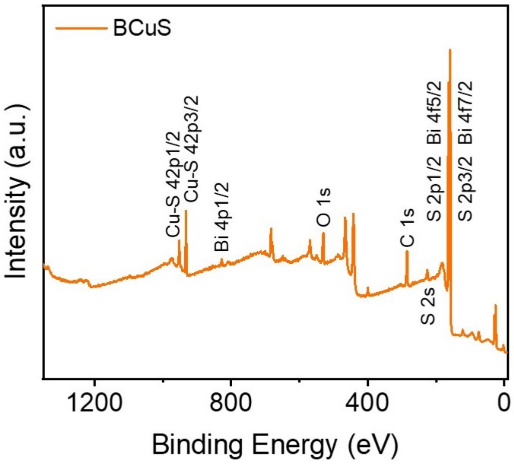

Figure S3. XPS survey spectrum of BCuS.

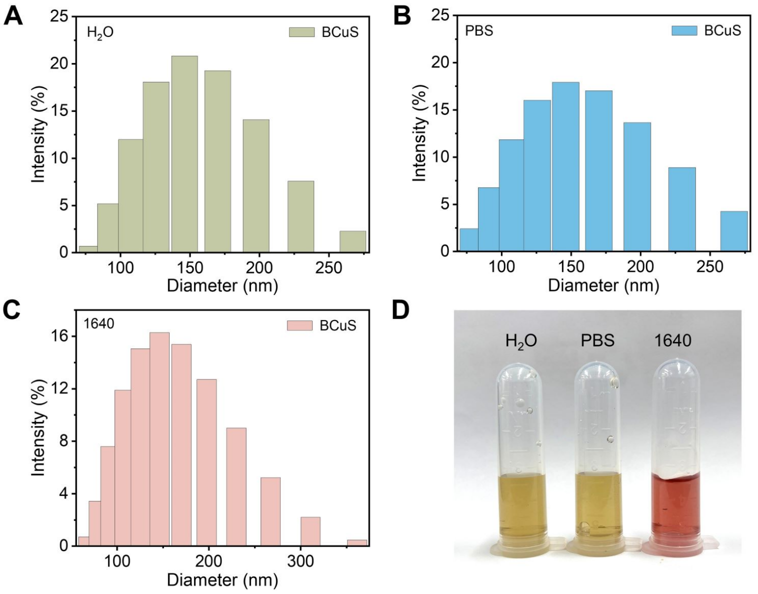

Figure S4. Hydrated particle size distribution of BBP in (A) water, (B) PBS, and (C) RPMI-1640. (D) Digital photographs of BCuS (100 μg mL^{-1}) in water, PBS, and RPMI-1640 culture medium.

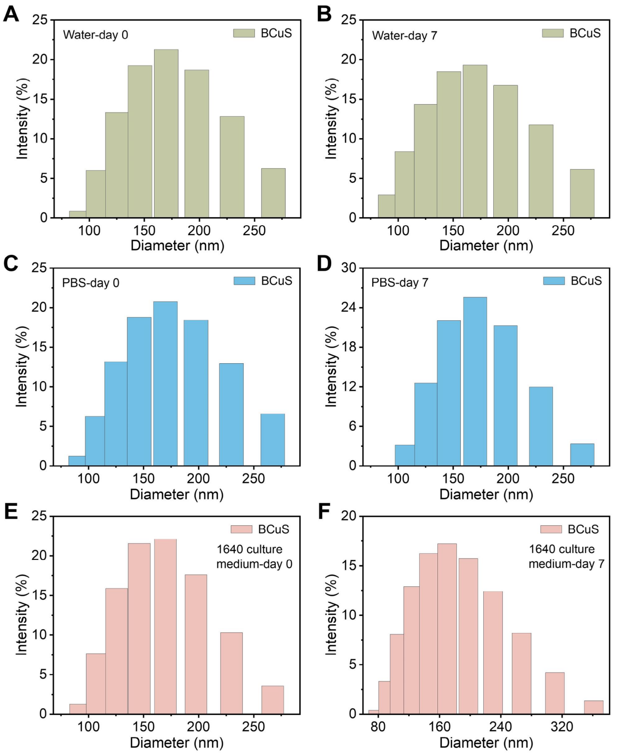

Figure S5. Hydrated particle size diagram of BCuS (100 μg mL^{-1}) in water, PBS, and RPMI-1640 culture medium. Hydrated particle size diagram of BCuS on (A) day 0 and (B) day 7 in water, (C) day 0 and (D) day 7 in PBS, (E) day 0 and (F) day 7 in 1640 culture medium.

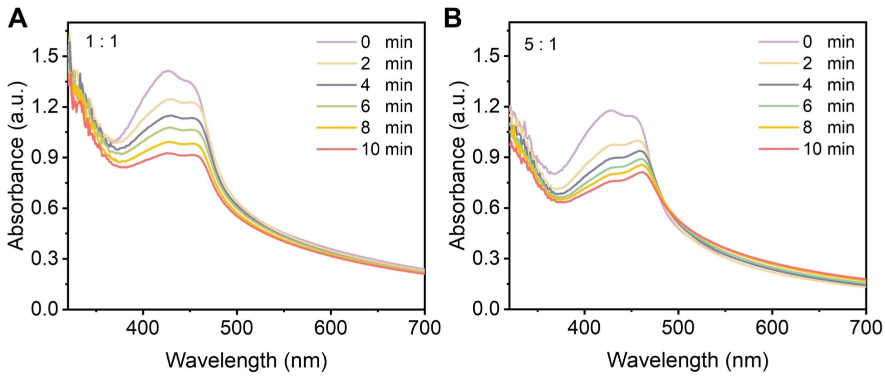

Figure S6. The absorption spectra of BCuS mixed with DPBF synthesized under the condition of BiF_3 and $Cu(NO_3)_2$ feeding ratio of (A) 1:1 and (B) 5:1 at different US times.

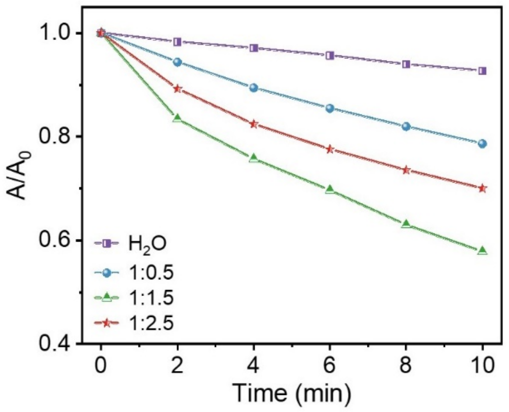

Figure S7. Time-dependent relative absorption changing at 421 nm of H_2O and BCuS with DPBF at different ultrasound times, among BCuS synthesized under different BiF_3 and $Cu(NO_3)_2$ feed ratios mixed, respectively.

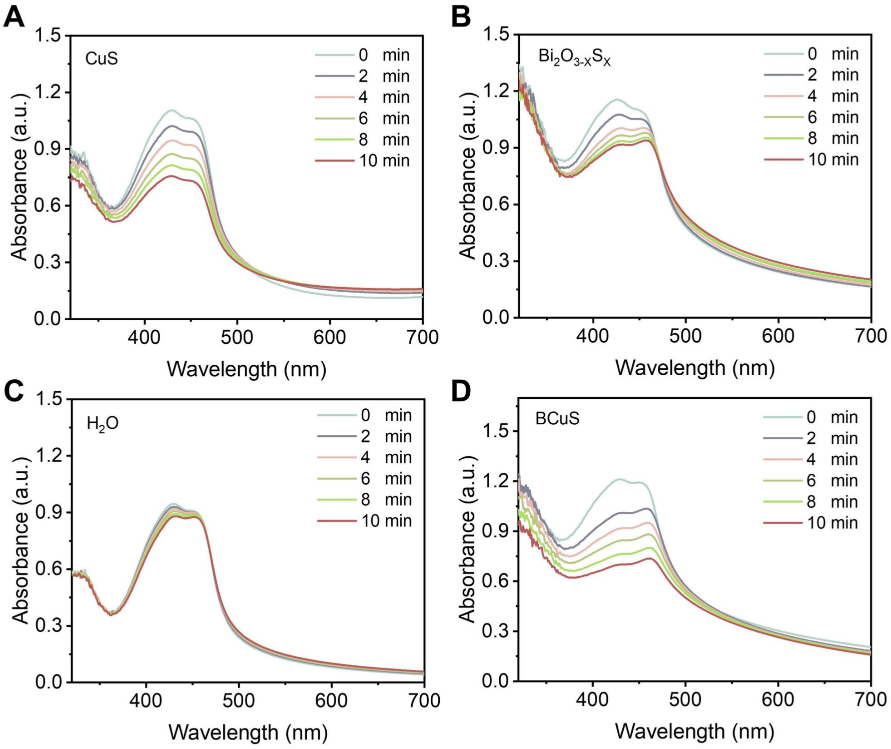

Figure S8. Time-dependent absorption spectra of DPBF in different groups mixed with (A) CuS, (B) $Bi_2O_{3-x}S_x$, (C) H_2O, and (D) BCuS under US stimulation (1.0 W cm^{-2}).

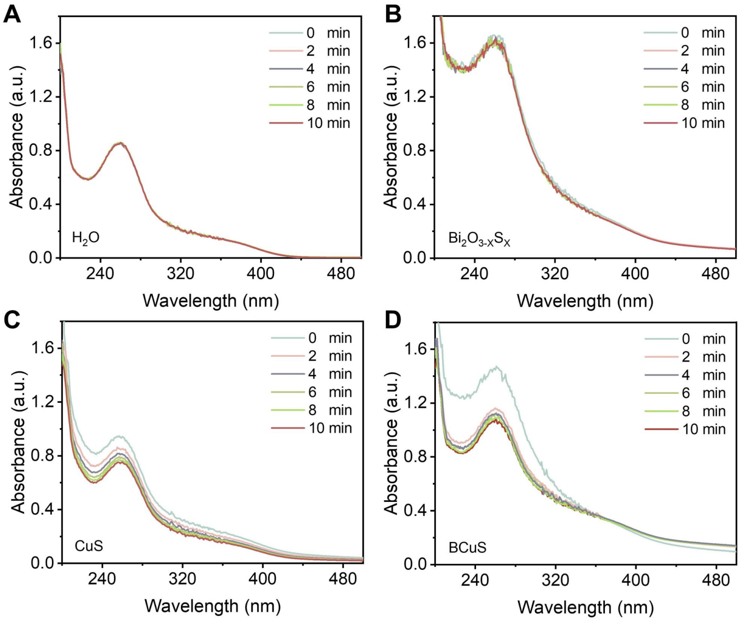

Figure S9. Time-dependent absorption spectra of DPBF in different groups mixed with (A) H_2O, (B) $Bi_2O_{3-X}S_X$, (C) CuS, and (D) BCuS under US stimulation (1.0 W cm^{-2}).

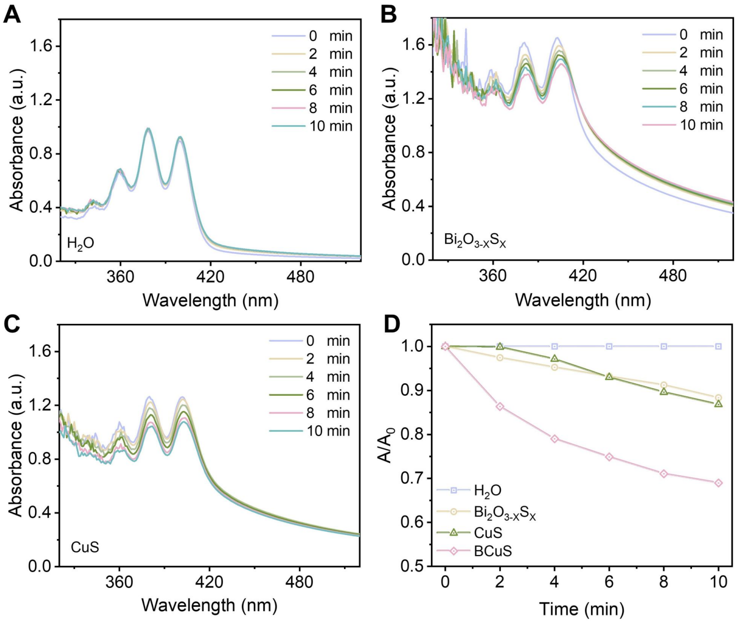

Figure S10. Time-dependent absorption spectra of DPA in different groups mixed with (A) H_2O, (B) $Bi_2O_{3-X}S_X$, and (C) CuS under US stimulation (1.0 W cm^{-2}). (D) Relative absorption changing at 400 nm in different groups over time.

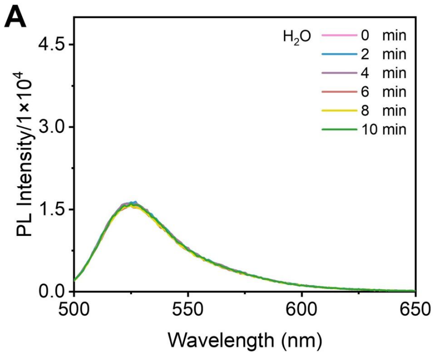

Figure S11. Absorption spectra of DHR123 in (A) H_2O and (B) BCuS under different US stimulation times (1.0 W cm^{-2}).

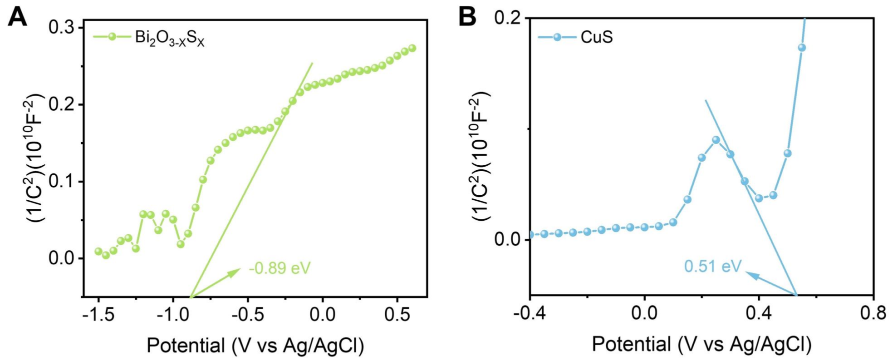

Figure S12. (A) Mott–Schottky plots of $Bi_2O_{3-x}S_x$. (B) Mott–Schottky plots of CuS.

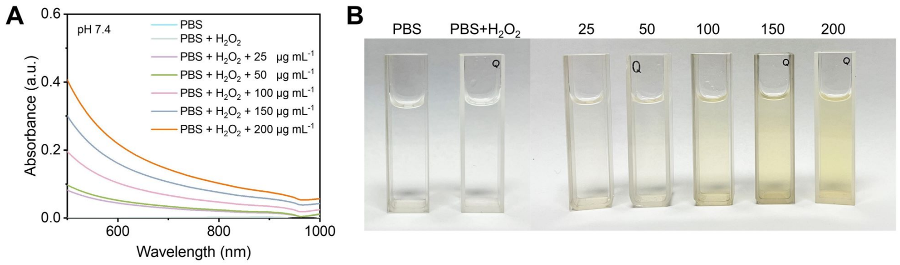

Figure S13. (A) Absorption spectra of ox-TMB in the mixed solution as a function of BCuS concentration under pH 7.4/H_2O_2 conditions. (B) Digital photographs showing the color change of ox-TMB under pH 7.4/H_2O_2 conditions.

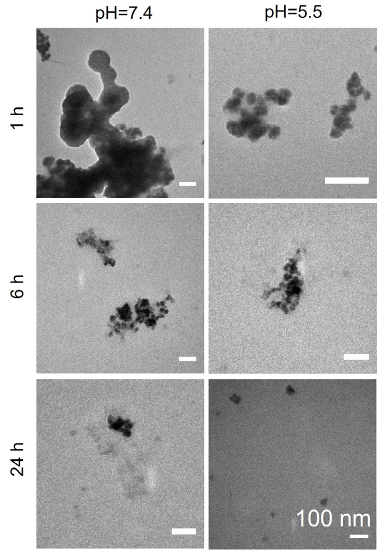

Figure S14. TEM images of BCuS after incubation for different times under pH 5.5/GSH and pH 7.4/GSH conditions.

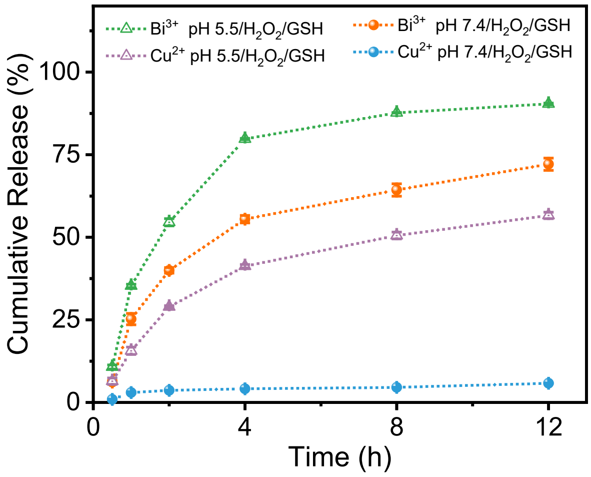

Figure S15. Accumulation of Cu^{2+} and Bi^{3+} in solution under H_2O_2/GSH (pH 7.4 or 5.5) conditions detected by ICP-OES.

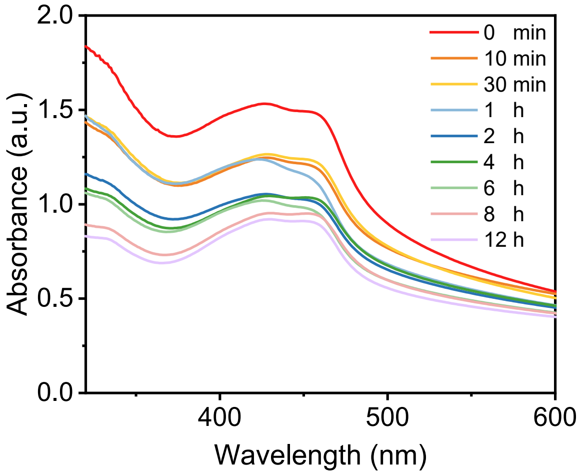

Figure S16. Absorption spectra of BCuS and DPBF solutions incubated with GSH at different times under US stimulation for 5 min.

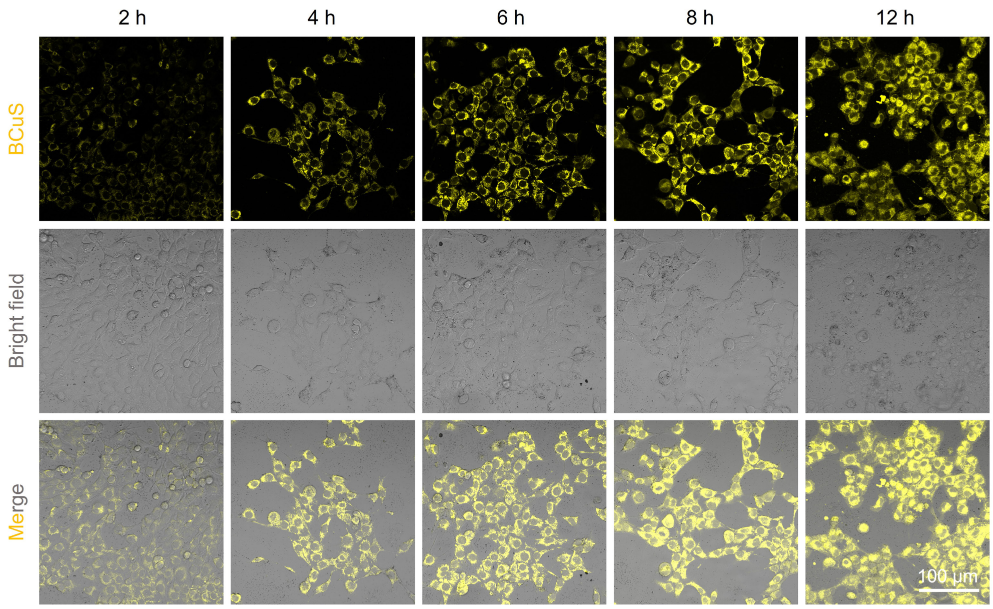

Figure S17. Cell fluorescence images of RhB-labeled BCuS over different incubation times.

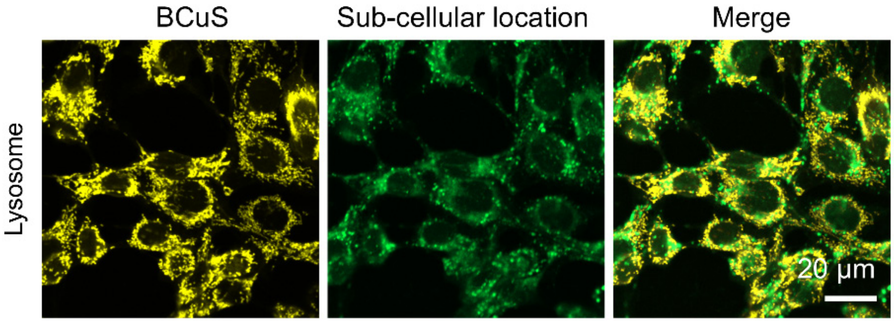

Figure S18. Co-localization of BCuS-RhB in lysosome.

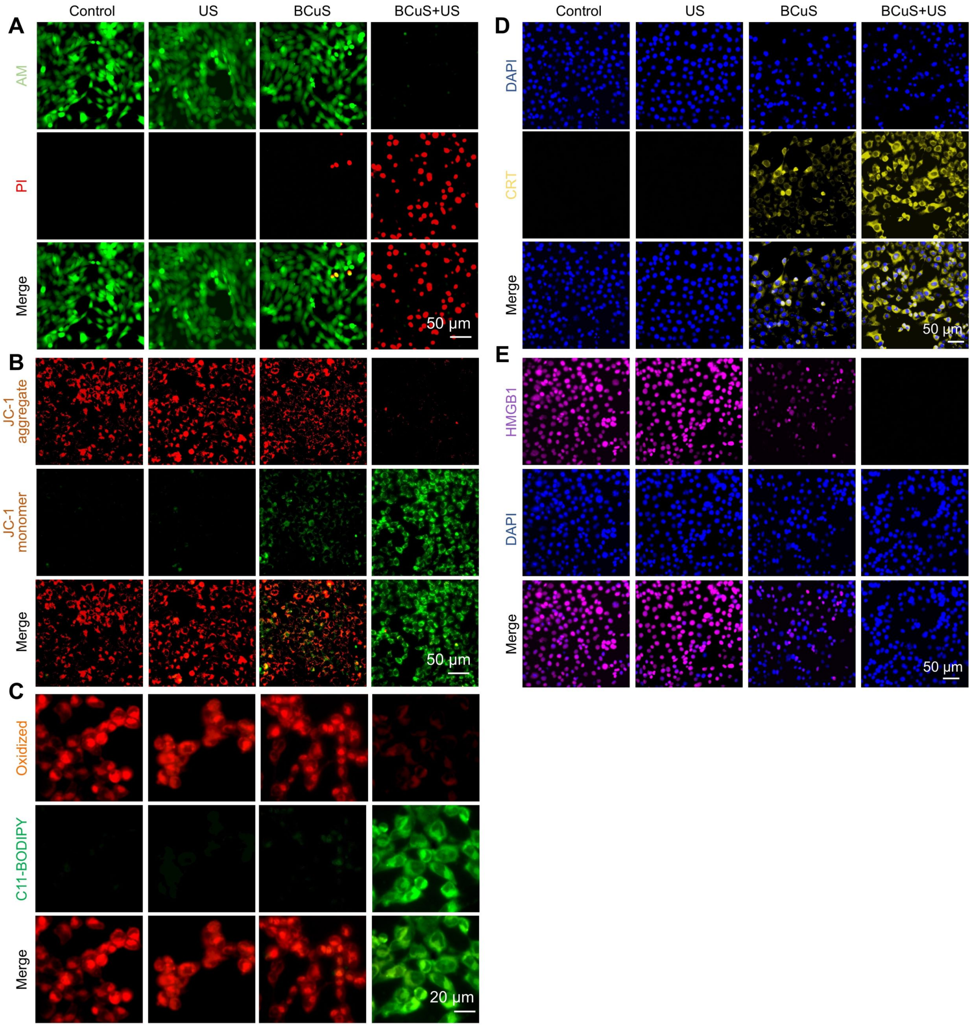

Figure S19. (A) Calcein-AM/PI, (B) JC-1, (C) C11-BODIPY, (D) CRT, and (E) HMGB1 staining images of 4T1 cells in different treatment groups.

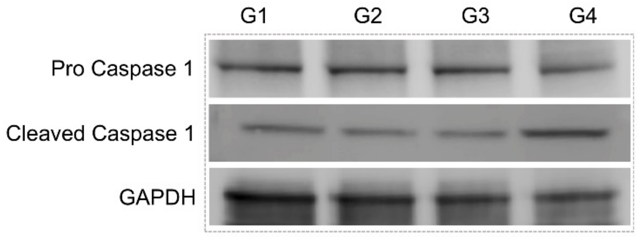

Figure S20. WB analysis of Pro Caspase 1 and Cleaved Caspase 1 in 4T1 cells in different treatment groups. (G1) control, (G2) US, (G3) BCuS, and (G4) BCuS+US.

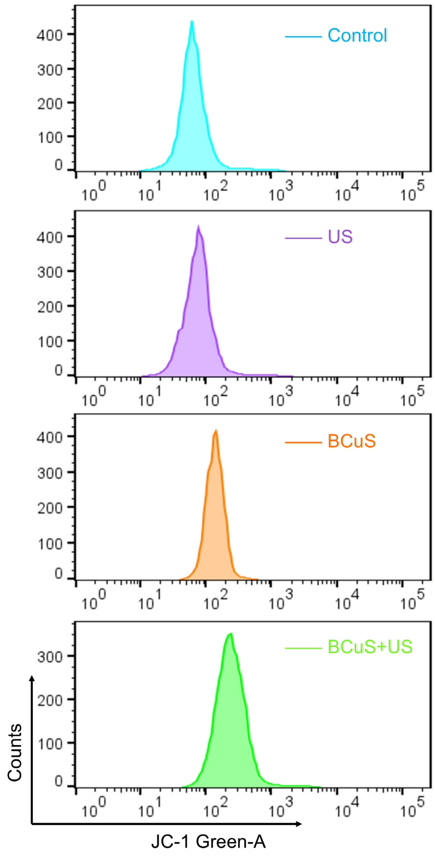

Figure S21. Flow cytometry analysis of JC-1-stained cells in different treatment groups.

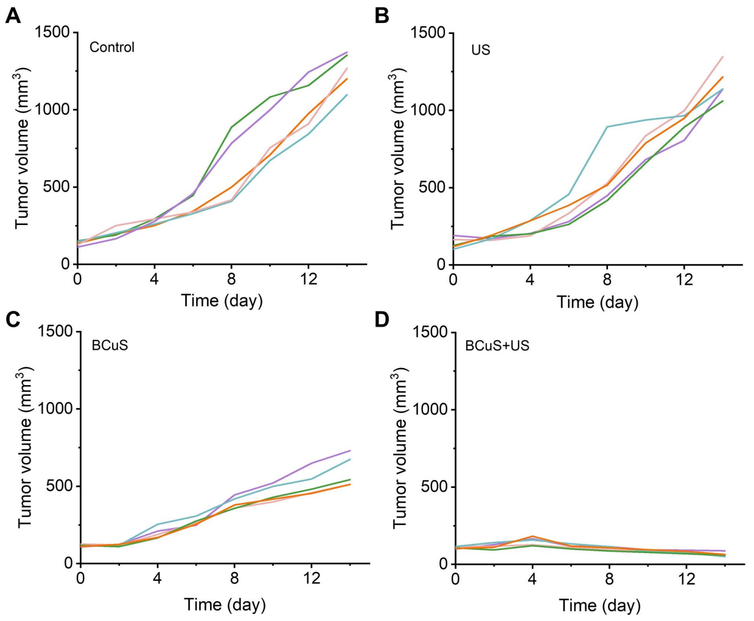

Figure S22. The individual growth curves of 4T1 primary tumors in different treatment groups (A) control, (B) US, (C) BCuS, and (D) BCuS+US.

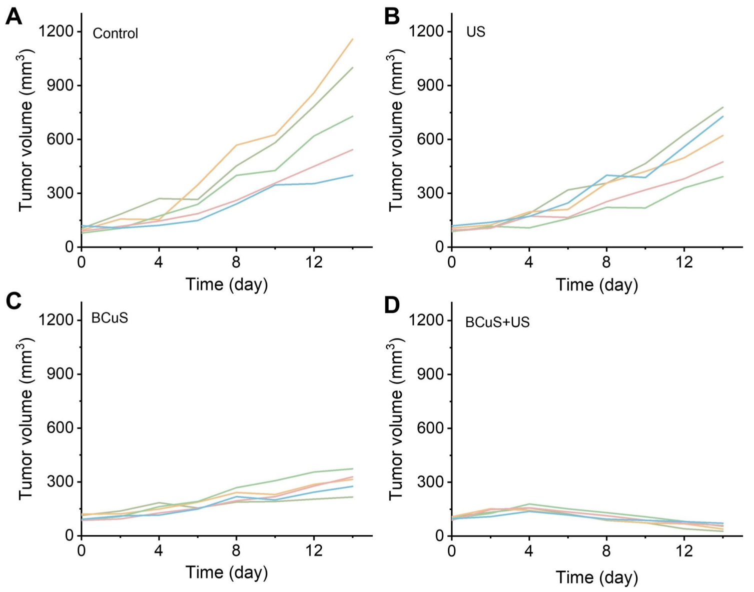

Figure S23. The individual growth curves of 4T1 distant tumors in different treatment groups (A) control, (B) US, (C) BCuS, and (D) BCuS+US.

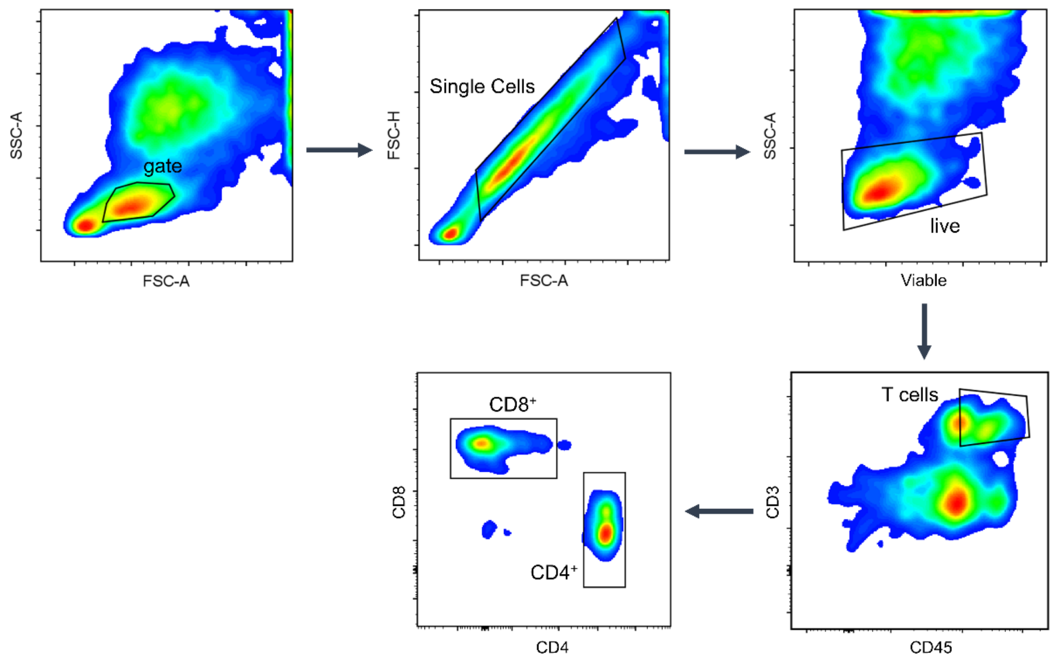

Figure S24. Gating strategy of FACS analysis for measuring CD8$^+$ cells and CD4$^+$ cells in the CD3$^+$ cells.

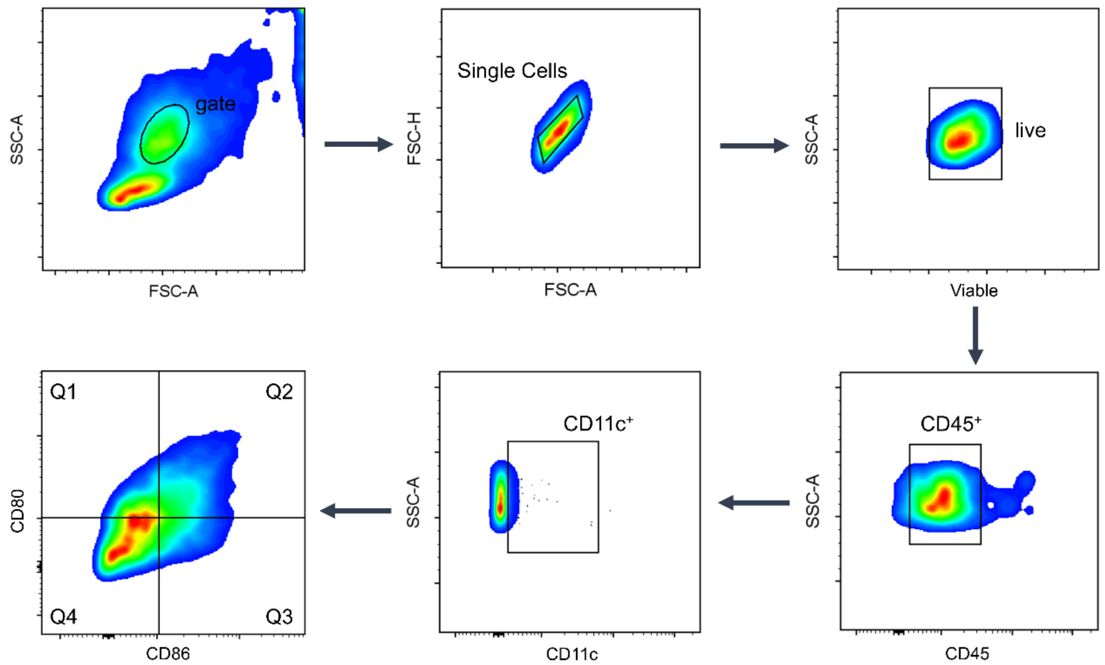

Figure S25. Gating strategy of FACS analysis for measuring mature DCs in the CD11c$^+$ cells.

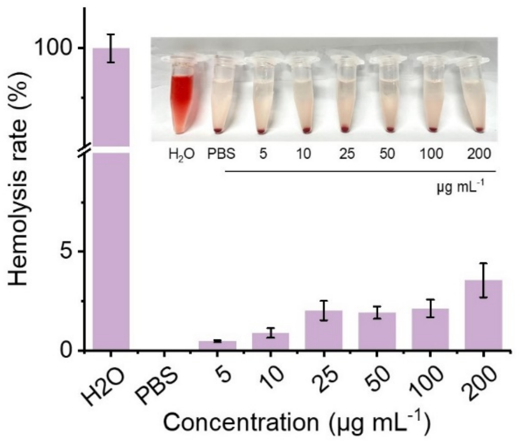

Figure S26. Hemolytic analysis of BCuS with various concentrations. The inset are bright field photos for each group.

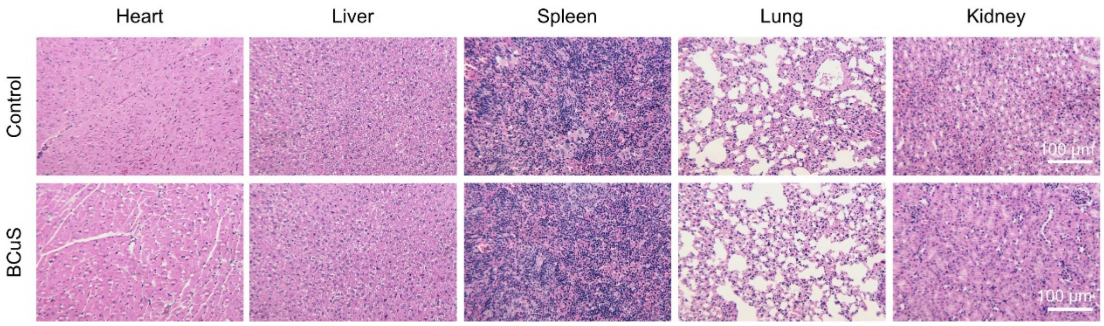

Figure S27. H&E-stained sections of mice major organs in the control group and mice after BCuS injection with US treatment 15 days.

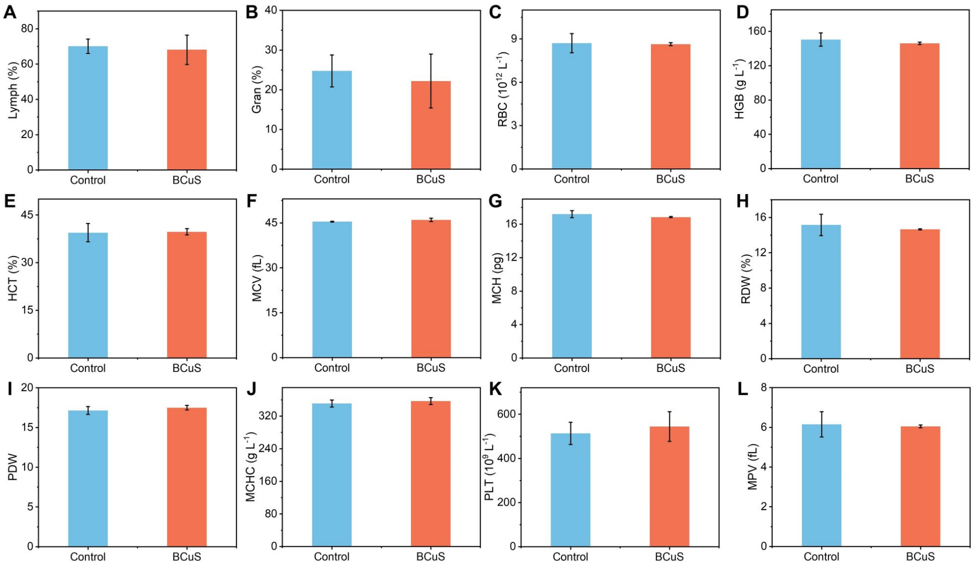

Figure S28. Blood routine standard data of mice in the control group and mice after BCuS injection with US treatment 15 days. (A) lymphocyte (Lymph). (B) neutrophil (Gran). (C) Red blood cell (RBC). (D) Hemoglobin (HGB). (E) Plateletocrit (PCT). (F) Mean corpuscular volume (MCV). (G) Mean corpuscular hemoglobin (MCH). (H) Red blood cell distribution (RDW). (I) Platelet distribution width (PDW). (J) Mean corpuscular hemoglobin concentration (MCHC). (K) Platelet (PLT). (L) Mean platelet volume (MPV).

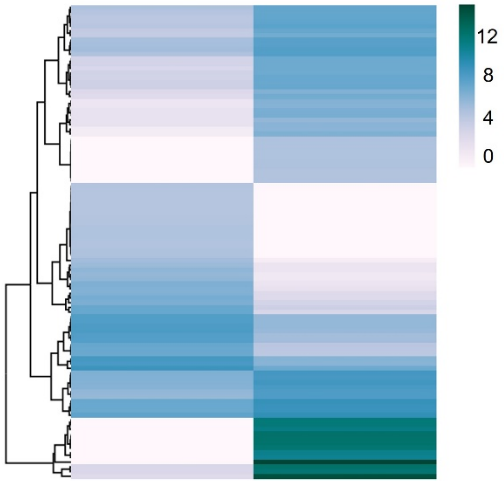

Figure S29. Heatmap of DEGs.
